# Further Insights Into the Metabolism of LGD‐4033 in Human Urine. Part 1. Structure Elucidation of Additional Important Metabolites

**DOI:** 10.1002/dta.70009

**Published:** 2025-12-09

**Authors:** Yiannis S. Angelis, Panagiotis Sakellariou, Mario Thevis, Andreas Thomas, Michael Petrou, Emmanuel N. Pitsinos

**Affiliations:** ^1^ Institute of Biosciences & Applications, National Centre for Scientific Research “DEMOKRITOS”, Doping Control Laboratory of Athens Athens Greece; ^2^ Institute of Biochemistry/Center for Preventive Doping Research German Sport University Cologne Cologne Germany; ^3^ European Monitoring Center for Emerging Doping Agents Cologne Germany; ^4^ Cyprus Anti‐Doping Authority Nicosia Cyprus; ^5^ Institute of Nanoscience and Nanotechnology, National Centre for Scientific Research “DEMOKRITOS” Athens Greece; ^6^ Department of Chemistry National and Kapodistrian University of Athens Athens Greece

**Keywords:** doping, LC‐HRMS/MS, LGD‐4033 metabolism, organic synthesis, structure elucidation

## Abstract

This study presents LC‐HRMS/MS analyses of LGD‐4033 post‐administration urine samples, following hydrolysis with *β*‐glucuronidase and liquid–liquid extraction, against chemically synthesized molecules that matched previously proposed metabolites, characterized by ^1^H and ^13^C NMR. Using this targeted metabolic investigation approach and the direct comparison of retention times and mass spectral data (high‐resolution full scan mass accuracy and collision‐induced fragmentation patterns), in accordance with WADA's TD2023IDCR provisions, resulted in unambiguous structural elucidation of additional LGD‐4033 metabolites, including (a) the *epi*‐long‐term dihydroxylated metabolite (**M5a**); (b) the *epi*‐pyrrolidinone metabolite (**M2d**); (c) the (*R*,*R*)‐diastereoisomer of the ring‐opened hydroxylated metabolite (**M4b**); and (d) one of the two detected tris‐hydroxylated metabolites (**M6a**). Additionally, a new, previously undescribed metabolite, which is a hydroxylated derivative of the pyrrolidinone metabolite **M2c**, was also detected up to 4 days post‐administration and coded as **M7**. Metabolites **M5a** and **M2d** are detectable up to 21 days post‐administration and can be considered additional long‐term markers. These findings expand current knowledge of LGD‐4033 metabolism. From a doping control perspective, the proposed synthetic pathways may facilitate the production of reference materials for the detection and identification of a more comprehensive metabolite profile that will increase metabolic certainty in future LGD‐4033 adverse analytical findings.

## Graphical abstract

Well‐characterized, chemically synthesized molecules related to LGD‐4033 metabolism were used as analytical standards for the analysis of LGD‐4033 post‐administration urine samples. This approach offered further insights into LGD‐4033 metabolism, providing solid experimental proof for the structure of important previously reported metabolites and facilitating the identification of a new one.

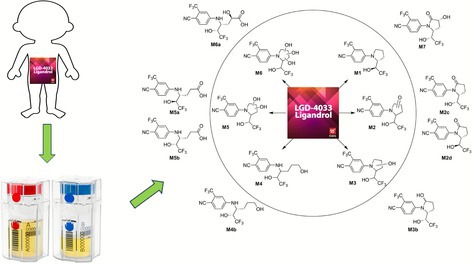

## 1 Introduction

**FIGURE 1:**
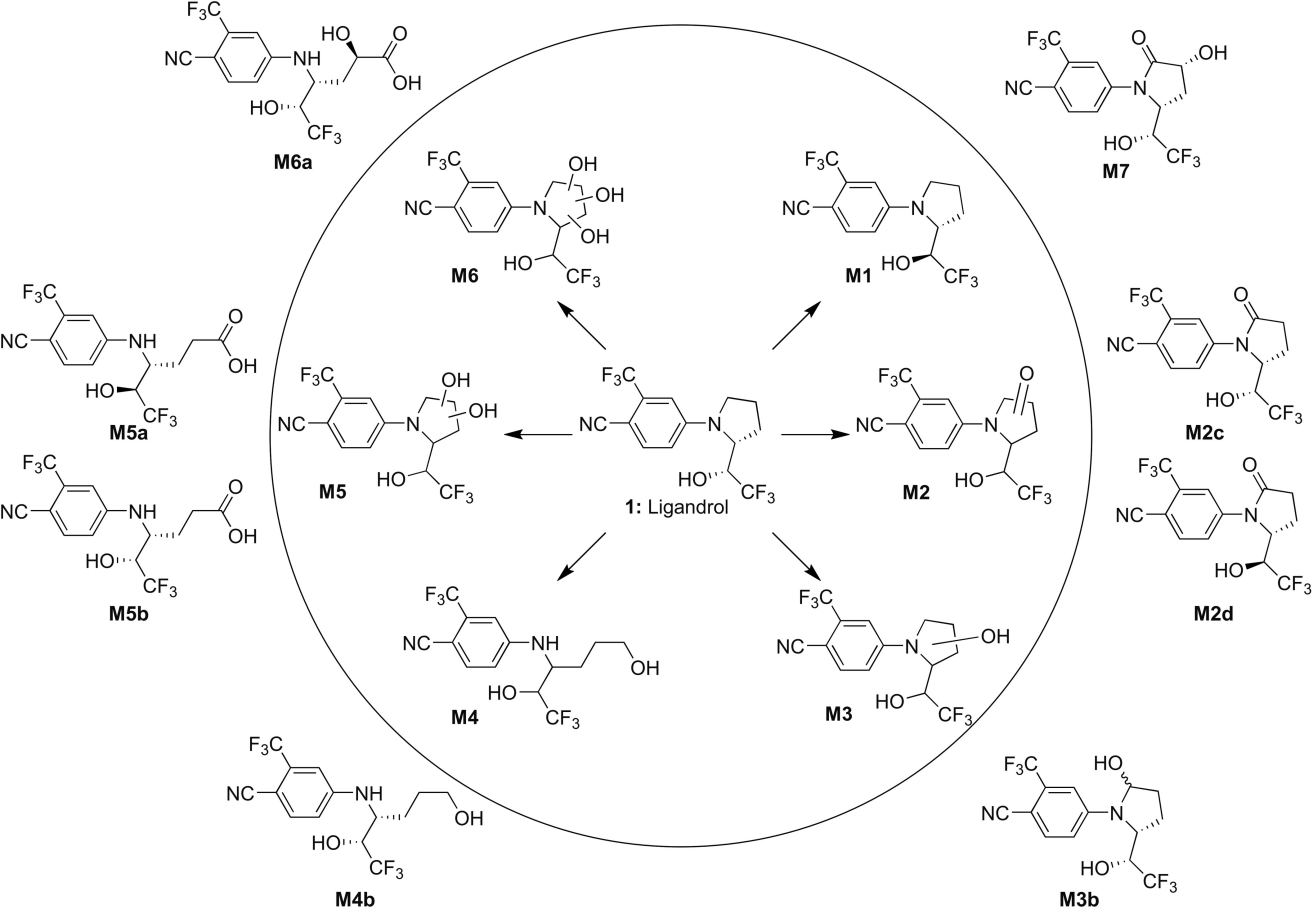
General (inside circle) and refined (periphery) structures of pyrrolidine ring‐related metabolites of LGD‐4033. The metabolite coding follows that presented by Wagener et al. [5].

LGD‐4033 (also known as Ligandrol) is a non‐steroidal Selective Androgen Receptor Modulator (SARM) that is frequently detected during sports drug testing [1], despite its inclusion in the prohibited list [2] of the World Anti‐Doping Agency (WADA), due to its promising anabolic effects [3, 4]. It is extensively metabolized in the human body and the general structures of its long‐term metabolites (**M1**–**M6**, Figure 1) have been described in the literature [6, 7, 8, 9, 10]. As part of our continued interest in the metabolism of substances related to sports doping, we were able to fully elucidate the structure of the main long‐term metabolite of LGD‐4033 (**M5b**, Figure 1) [11], through comparison with well‐characterized synthetic standards, and to reveal the potential of a pyrrolidone‐type metabolite (**M2c**, Figure 1) as a new long‐lasting metabolite [12]. For these long‐term metabolites, isobaric peaks that display similar product ion mass spectra are typically detected in post‐administration samples. These isobaric peaks have been tentatively attributed to epimers of the LGD‐4033 long‐term metabolites. However, solid experimental proof for their structures, such as comparison of their retention times and product ion mass spectra with synthetic standards, is missing.

Earlier efforts in the metabolism of LGD‐4033 [11, 12] suggest that missing metabolic markers might correspond to potential intermediates or extensions of the biosynthetic pathway that metabolically converts the parent LGD‐4033 into the already fully characterized long‐term metabolites. To assess this hypothesis, the epimers of metabolites **M2c** and **M5b** (Figure 1) were synthesized, starting from the commercially available LGD‐4033 epimer, in analogy to the previously reported synthetic pathway [11]. Furthermore, additional envisioned structures, like **M4b** and **M6a** (Figure 1), that could correspond to previously proposed metabolites of LGD‐4033 [6, 7, 8, 9, 10], were also targeted synthetically. Post‐administration samples collected after the ingestion of a supplement containing, according to the labe, 10 mg of LGD‐4033 were analyzed along with chemically synthesized, well‐characterized standards by liquid chromatography‐high resolution (tandem) mass spectrometry (LC‐HRMS/[MS]) under full scan and parallel reaction monitoring (PRM) mode. Retention times and product ion ratios were then evaluated under the provisions of WADA TD2023IDCR [13]. Using this targeted metabolic investigation approach, experimental proof was secured and is presented in this study for the structural elucidation of additional metabolites of this popular among cheaters SARM. Additionally, using a synthetic intermediate as standard, a new, previously undescribed metabolite, namely, an *α*‐hydroxylated derivative of metabolite **M2c**, coded as **M7**, was detected. An updated, detailed scheme of the metabolism of LGD‐4033 is shown in Figure 1.

## 2 Materials and Methods

Detailed information regarding the synthesis of the compounds and the materials used during this study is provided in the Supporting Information.

### 2.1 Samples

Urine samples obtained in a previous administration study [10], which was approved by the National Bioethics Committee of Cyprus (Decision number: EEBK 21.1.01.03/21.04.2017) were used in the present study with the approval of the Bioethics Committee. Urine samples were obtained after the administration of the supplement Ligandrol (purchased from Neobolics, Montreal, Canada) to one healthy, human male volunteer (Caucasian, 48 years old, 60 kg): one capsule × 10 mg of LGD‐4033. The urine samples were collected before (0 h) and after administration up to 494 h (almost 21 days). The exact time intervals (h) were as follows: 0, 2, 4, 4.5, 8, 10.5, 12, 15, 20, 24, 36, 48, 60, 72, 84, 96, 108, 120, 135, 145.5, 156.5, 168, 180, 193, 204, 216, 228, 240, 252, 264, 279, 288, 300, 312, 324, 337, 349, 361, 373, 385, 397, 409, 420.5, 432, 445, 457, 468.5, 481, and 494. All collected samples were kept frozen at −20°C until analysis.

### 2.2 Sample Preparation Procedures

For the extraction of LGD‐4033 metabolites a common ITP procedure for the detection of doping substances using LC‐HRMS was used comprised of the addition of a mixture of internal standards (ISTDs), including dexamethasone‐d4 (30 ng/mL), ephedrine‐d3 (50 ng/mL), fluticasone propionate‐d5 (30 ng/mL), formoterol‐d6 (20 ng/mL), furosemide‐d5 (200 ng/mL), nalburphine (25 ng/mL), propranolol‐d7 (50 ng/mL), testosterone‐d3 (10 ng/mL), and trans‐11Nor‐Δ9‐THC‐COOH‐d3 (10 ng/mL), to 3 mL of urine samples. The sample pH was adjusted to 7.0 using phosphate buffer 1 M and then 30‐μL *β*‐glucuronidase from *Escherichia coli* was added. Urine samples were incubated for 1.5 h at 55°C. Following hydrolysis, urine pH was adjusted to 9.5 with NaHCO_3_:Na_2_CO_3_ (10:1, w/w) solid buffer and hydrolates were extracted with 5 mL of ethyl acetate after the addition of 3 g of anhydrous Na_2_SO_4_ for salting out. Samples were centrifuged and then the organic layer was transferred to glass tubes; the mixture was evaporated to dryness under a stream of nitrogen at 60°C. Dry residues were reconstituted with 100 μL of a 50% mixture of acetonitrile in water and transferred to 1.5‐mL Eppendorf tubes. Then, 100 μL of 5‐mM ammonium formate in 0.02% formic acid was added, and samples were centrifuged for 10 min at 10,000 rpm. After the centrifuge, 150 μL were transferred to vials with inserts. Twenty microliters of the sample were injected in LC‐HRMS/MS without any further purification.

### 2.3 LC‐HRMS/(MS) Analysis

A Dionex UHPLC system (Thermo Scientific, Bremen, Germany) was used for the chromatographic separation. The system consisted of a vacuum degasser, a high‐pressure binary pump, an autosampler with a temperature‐controlled sample tray set at 7°C, and a column oven set at 30°C. Chromatographic separation was performed at 30°C using a Zorbax Eclipse Plus C18 column (100 × 2.1 mm i.d., 1.8‐μm particle size; Agilent Technologies). The mobile phase consisted of 5 mM ammonium formate in 0.02% formic acid (Solvent A) and a mixture of acetonitrile/water (90:10, v/v) containing 5 mM ammonium formate and 0.02% formic acid (Solvent B). A gradient elution program was employed at a constant flow rate of 0.2 mL/min with solvent B starting at 5% for 3 min, initially increasing to 30% in 4 min, then increasing to 90% in 11 min and finally, set back to 5% in 11.5 min. The post‐run equilibrium time was 3.5 min. The injection volume was 3 μL. A QExactive benchtop Orbitrap‐based mass spectrometer (ThermoScientific, Bremen, Germany) equipped with a heated electro‐spray ionization (HESI) source operated in negative polarity mode in full scan from *m*/*z* 100–1000 at 17,500 resolving power and injection time of 100 ms. Source parameters were: sheath gas (nitrogen) flow rate, auxiliary gas (nitrogen) flow rate and sweep gas flow rate: 40, 10, and 1 arbitrary units, respectively; capillary temperature: 300°C; ESI heater temperature: 30°C; spray voltage: +4.0 kV. For the structural investigation of metabolites LC‐HRMS product ion scans were performed for the deprotonated molecules at selected ions with an isolation window of *m*/*z* 1.0 at 17,500 resolving power in various collision energies and injection time of 100 ms (product ion mode). The automatic gain control (AGC) was set at 10E6 ions. The mass calibration of the Orbitrap instrument was evaluated in both positive and negative modes weekly and external calibration was performed prior to use following the manufacturer's calibration protocol.

## 3 Results and Discussion

### 3.1 Metabolites **M5a** and **M5b**

**FIGURE 2:**
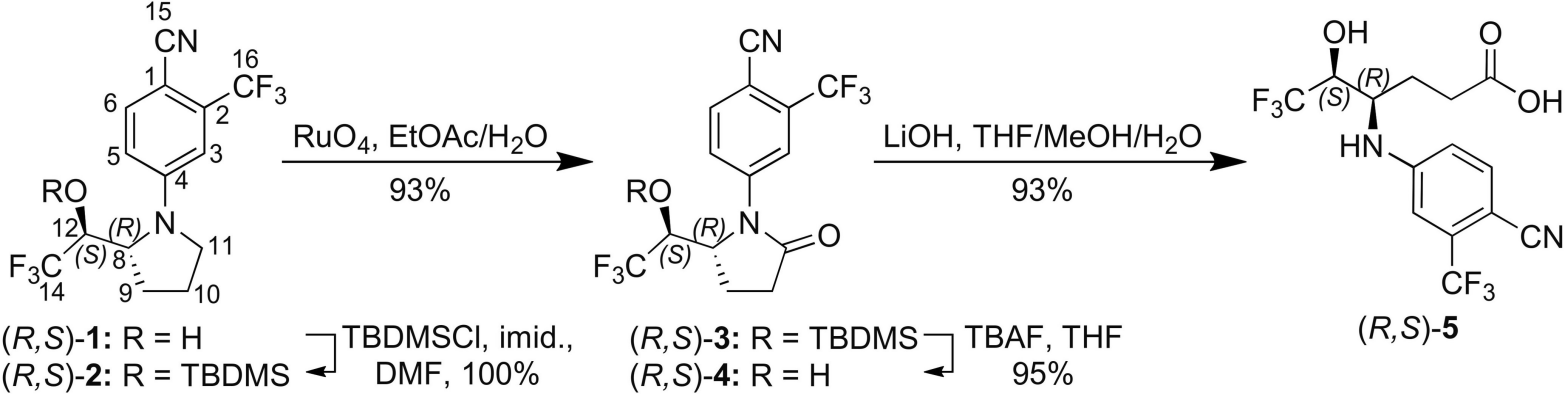
Synthesis of (4*R*,5*S*)‐**5** from 12‐*epi*‐LGD‐4033.

The main long‐term metabolite of LGD‐4033 (**M5b**, Figure 1) is commonly the metabolite of choice for monitoring its illicit use by WADA‐accredited doping control laboratories, and its synthesis and structure elucidation were achieved by our group [11]. Due to their very similar retention times (Δ*R*_*t*_ = 0.1–0.2 min) and identical molecular ion *m/z*, WADA‐accredited laboratories simultaneously monitor both the main (**M5b**) and the minor (**M5a**) isomeric metabolites of LGD‐4033 in their initial testing procedure (ITP). Furthermore, **M5a** itself is a long‐term metabolite, as it can be detected for nearly as long as **M5b** in controlled administration studies with low doses of LGD‐4033 that mimic contamination scenarios [5]. Interestingly, in one case where a single dose of 10 μg was administered to five volunteers, **M5a** was detected even longer than **M5b** [5]. On the other hand, in controlled administration studies with multiple microdoses of LGD‐4033, **M5a** elimination was faster than that of **M5b** [5]. Although the structure indicated in Figure 1 has been assigned to **M5a**, it was not rigorously established: It was proposed that **M5a** could be the epimer of **M5b**, due to the presence of *epi*‐LGD‐4033 (**M1**) in post‐administration urine samples and based on the similarity of **M5a** and **M5b** product ion mass spectra [5]. Consequently, the synthesis of the epimer of **M5b** and its evaluation as reference material for **M5a** were deemed relevant to doping controls and were, consequently, prioritized. To this end, the “chemo‐metabolic” approach employed for the synthesis of **M5b** from LGD‐4033 was adapted for the synthesis of **M5a** from commercially available *epi*‐LGD‐4033 [11]. Thus, in preparation for the subsequent regioselective oxidation of the pyrrolidine ring, the hydroxyl group of 12‐*epi*‐LGD‐4033 ([*R*,*S*]‐**1**, Figure 2) was protected as the silyl ether (*R*,*S*)‐**2** (Figure 2), by employing *tert*‐butyldimethylsilyl chloride (TBDMSCl) and imidazole in dimethylformamide (DMF). Subsequently, the use of RuO_4_, generated in situ from RuCl_3_ and 10% aqueous NaIO_4_, allowed the regioselective oxidation of the pyrrolidine ring under two‐phase conditions (i.e., EtOAc/H_2_O) [14], furnishing in good yield (93%) pyrrolidinone (*R*,*S*)‐**3** (Figure 2). Removal of the silyl protective group converted pyrrolidinone **3** to the (*R*,*S*)‐isomer of hydroxy‐pyrrolidinone **4**. Subsequent alkaline hydrolysis of the lactam ring secured the (4*R*,5*S*)‐diastereoisomer of the main dihydroxylated long‐term metabolite [(*R*,*R*)‐**5**].

**FIGURE 3:**
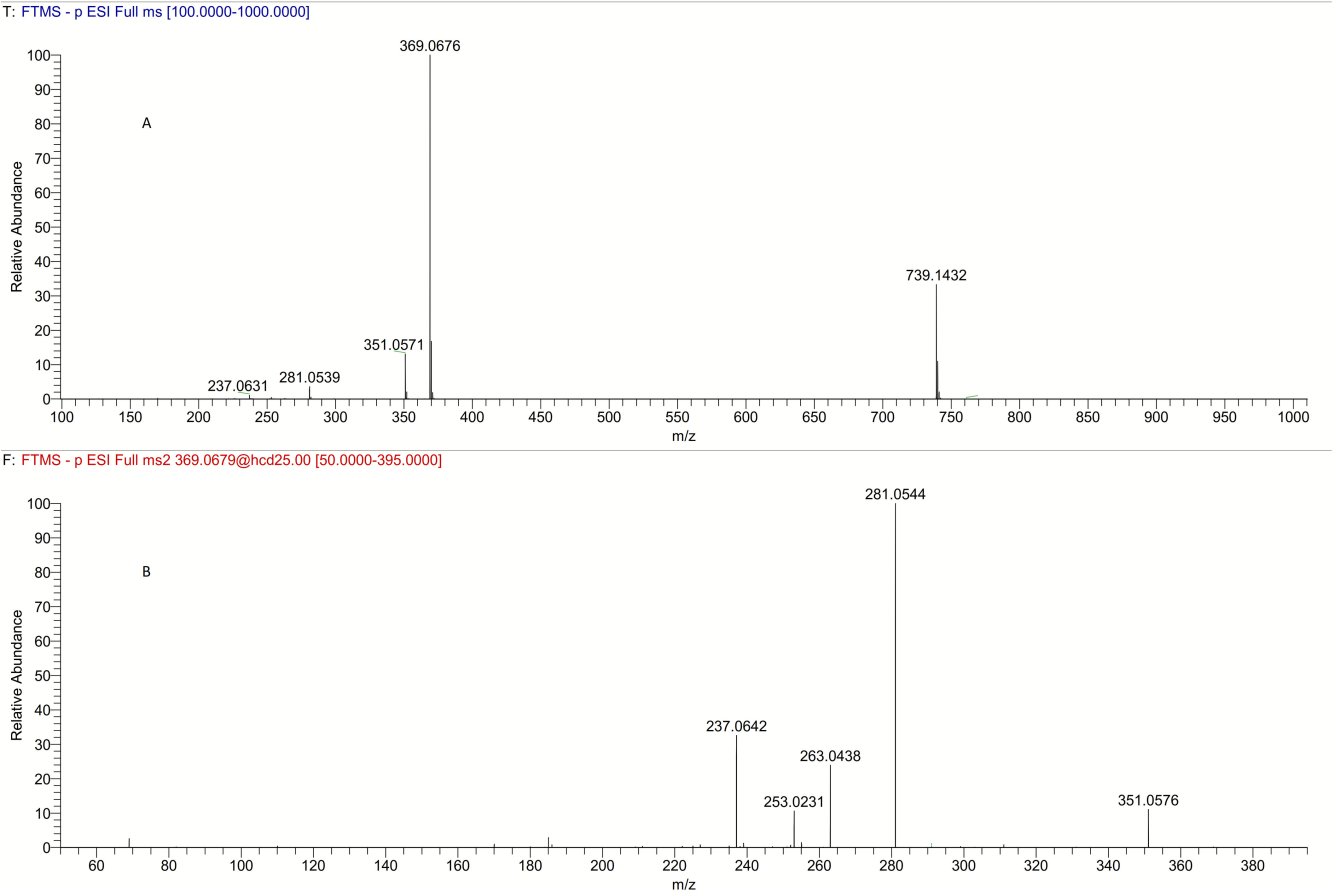
Full scan (A) and product ion (B) mass spectra of the (*R*,*S*)‐isomer of carboxylic acid **5** (synthetic standard).

**FIGURE 4:**
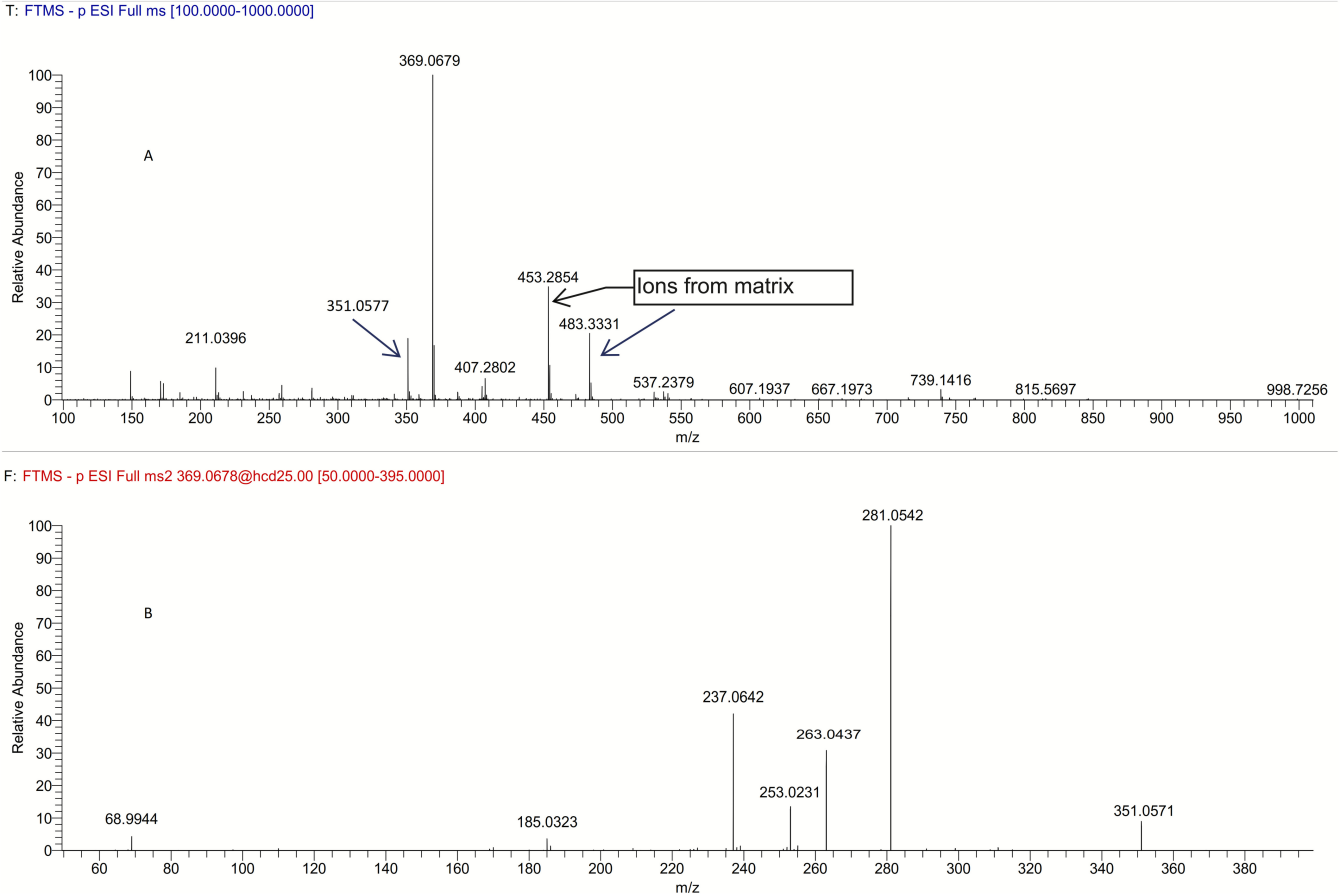
Full scan (A) and product ion (B) mass spectra of the metabolite **M5a** extracted from a post‐administration urine sample.

**FIGURE 5:**
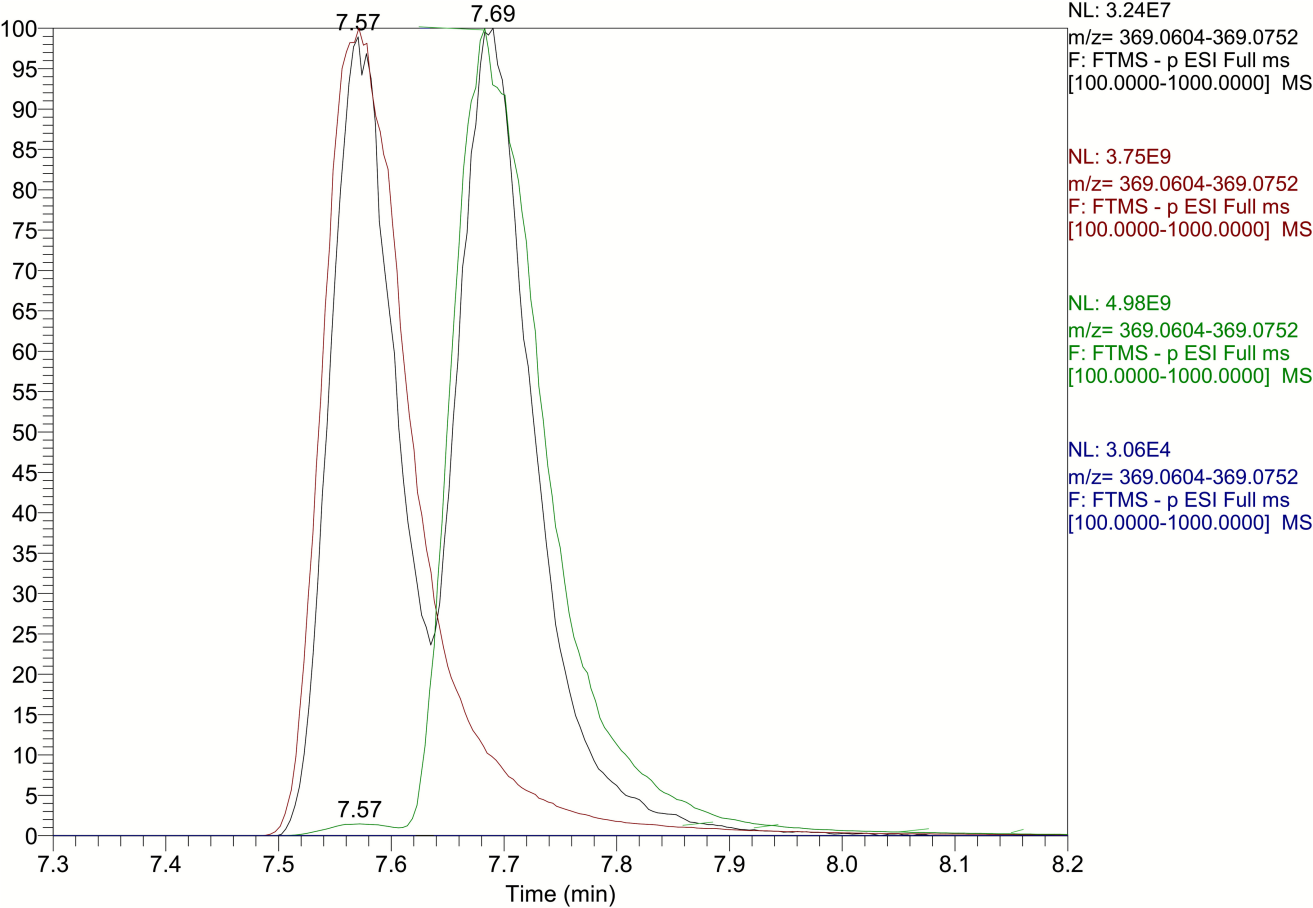
Extracted ion chromatograms at *m/*z 369.0679 [Μ‐Η]^−^ of a blank sample (in blue), a LGD‐4033 post‐administration urine sample (in black), and pure synthetic standards of the (*R*,*S*)‐isomer of carboxylic acid **5** (in red) and the (*R*,*R*)‐isomer of carboxylic acid **5** (in green).

In order to evaluate whether the synthesized carboxylic acid (*R*,*S*)‐**5** (Figure 2) corresponded to the minor metabolite **M5a** of LGD‐4033, its full scan and product ion mass spectra, obtained from electrospray ionization (ESI) in negative mode (Figure 3), were compared to those reported for the metabolite observed in urine (Figure 4) and were found to be identical. Furthermore, the retention time data (Figure 5) obtained from the LC‐HRMS analysis of synthetic (*R*,*S*)‐**5** against the minor long‐term metabolite of LGD‐4033 (**M5a**), which was extracted from a human urine sample obtained within the context of a previous related excretion study [10], deemed the two analytes identical based on WADA TD2023IDCR [13]. Thus, it was concluded that carboxylic acid (4*R,*5*S*)*‐* **5** is, in all respects examined, identical to the minor metabolite of LGD‐4033 (**M5a**) and could serve as reference material for this important metabolic marker.

### 3.2 Pyrrolidinone‐Type Metabolites (**M2**)

**FIGURE 6:**
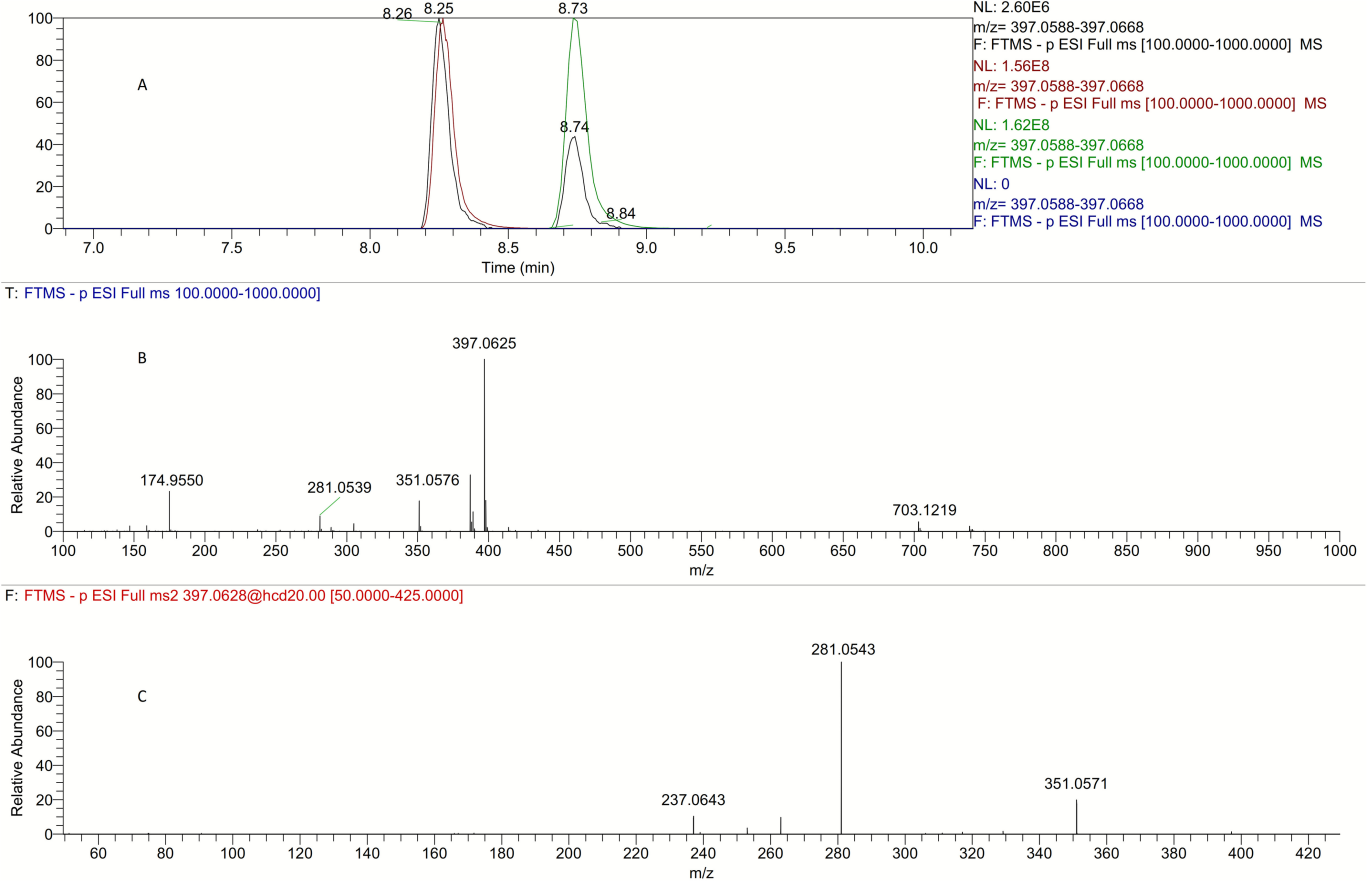
(A) Extracted ion chromatograms at *m/*z 397.0628 [M + FA‐H]^−^ of a blank sample (in blue), a post‐administration LGD‐4033 sample (in black) and pure synthetic (*R*,*R*)‐**4** (in red) and (*R*,*S*)‐**4** (in green), as standards for **M2c** and **M2d**, respectively. (B) Full scan mass spectrum of the (*R*,*S*)‐isomer of pyrrolidinone **4**, where the formate adduct at *m/z* 397.0628 [M + FA‐H]^−^ is the most abundant ion. (C) Product ion mass spectrum of the ion at *m/z* 397.0628.

Pyrrolidinone‐type metabolites (**M2**, Figure 1) with a theoretical [*M* − H]^−^ at *m/z* 351.0571 are detected in metabolism study samples with up to five chromatographic peaks [5]. Our previous study [12] showed that the first two signals of these deprotonated molecules correspond to dehydrative cyclization of the **M5a** and **M5b** metabolites during ESI (i.e., they are analytical artifacts) and that the third one corresponds to a genuine metabolite (**M2c**) featuring a pyrrolidinone ring where the keto group is located at the alpha position of LGD‐4033. The synthetic intermediate [*R*,*S*]‐**4** obtained during the synthesis of **M5a** (Figure 2) was tested against post‐administration urine samples and the results are presented in Figure 6. The most abundant ion in the mass spectra of both the (*R*,*S*)‐ and (*R,R*)‐isomers of pyrrolidinone **4** (as well as the metabolites **M2c** and **M2d**) is not the ion at *m/z* 351.0571, corresponding to the ion [*M* − H]^−^, but rather the formate adduct (FA) at *m/z* 397.0628 (Figure 6B) [12]. Thus, cross‐analysis of these peaks during the analysis of urine‐derived samples against pure synthetic standards of the (*R*,*S*) and (*R,R*)‐isomers of pyrrolidinone **4** at *m/*z 397.0628 confirmed that these peaks corresponded to these particular pyrrolidinone isomers (Figure 6A).

### 3.3 Monohydroxylated Metabolites (**M3** and **M4**)

**FIGURE 7:**
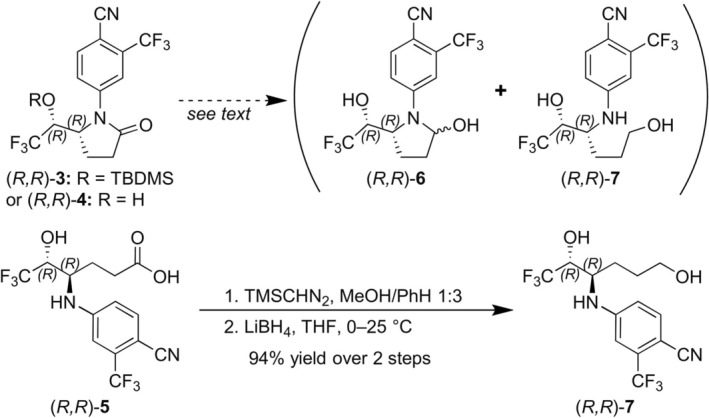
Synthesis of (*R*,*R*)‐**7**.

**FIGURE 8:**
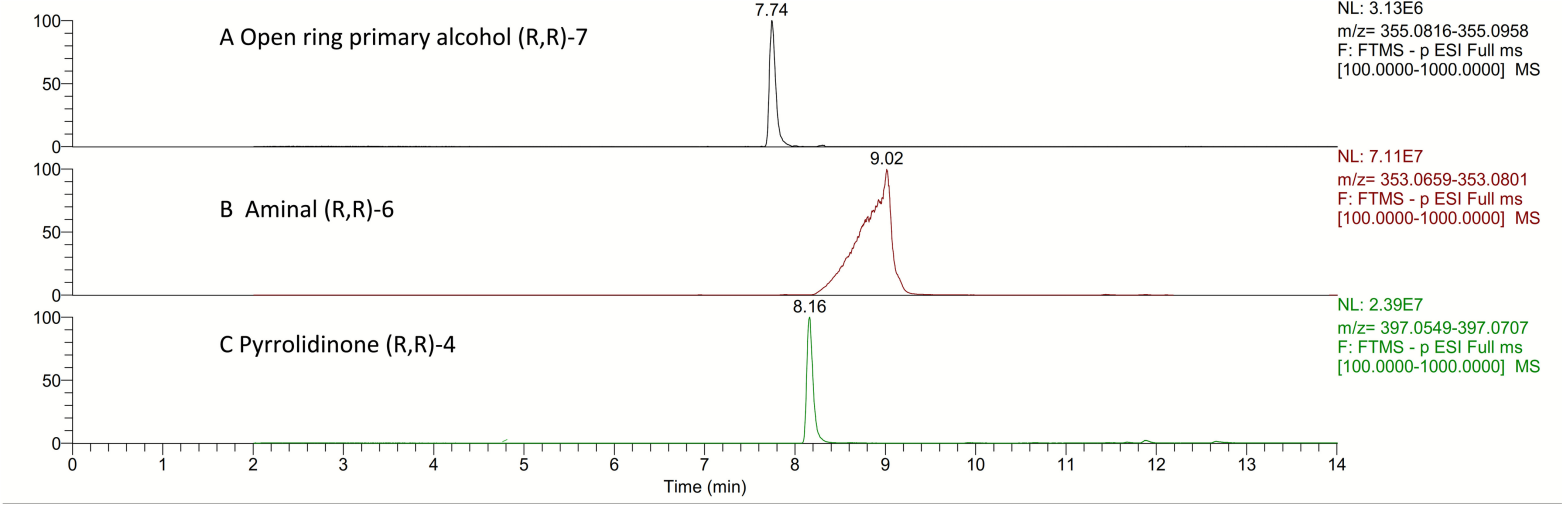
Extracted ion chromatograms of the crude reduction mixture of pyrrolidinone (*R*,*R*)‐**4** indicating the presence of (A) the open primary alcohol (*R*,*R*)‐**7** (Rt = 7.74 min; *m/z* 355.0887 [M‐H]^−^), (B) the desired aminal (4*R*,5*R*)‐**6** (Rt = 9.02 min; *m/z* 353.0730 [M‐H]^−^), and (C) the unreacted starting material (*R*,*R*)‐**4** (Rt = 8.16 min; *m/z* 397.0628 [M + FA‐H]^−^).

**FIGURE 9:**
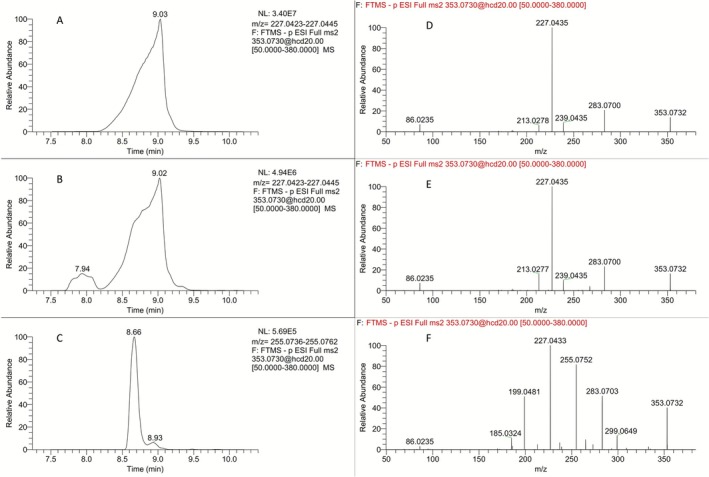
Extracted ion chromatograms for the ion transition of *m/z* 353.0730 [M‐H]^−^ to the product ion at *m/z* 227.0434 (**M3b**) of (A) a synthetic mixture and (B) a post‐administration sample extract. (C) extracted ion chromatogram of a post‐administration sample extract for the ion transition of *m/z* 353.0730 [M‐H]^−^ to the product ion at *m/z* 255.0749 corresponding to **M3a**. (D) Product ion mass spectrum of Peak A. (E) Product ion mass spectrum of Peak B. (F) Product ion mass spectrum of Peak C.

In the course of the synthesis of the (*R*,*R*)‐isomer of carboxylic acid **5**, the successful oxidation of the pyrrolidine ring of TBDMS‐protected LGD‐4033 to the corresponding pyrrolidinone (*R*,*R*)‐**3** (Figure 7) suggested the possibility of securing the diastereoisomeric aminals (i.e., pyrrolidines featuring a hydroxyl group located at the *α*‐position to the pyrrolidine nitrogen), some of which are reported [6] to correspond to the monohydroxylated metabolite **M3b** (Figure 1), through reduction of (*R*,*R*)‐pyrrolidinones **3** or **4** (Figure 7). However, when reduction of the silyl ether‐protected (*R*,*R*)‐pyrrolidinone **3** was attempted (LiBH_4_, THF, −78°C), although ^1^H NMR analysis of the partially purified product indicated it could contain the silyl ether derivative of the desired aminal‐type metabolite **M3b**, it was not possible to isolate it in pure form. Furthermore, the attempted removal of the silyl ether protective group (TBAF, THF), employing the partially purified reduction product, led to an even more complex mixture. To circumvent this obstacle, the free hydroxy‐pyrrolidinone (*R*,*R*)‐**4** (Figure 7) was subjected to reduction by DIBAL‐H (THF, −78°C). LC‐HRMS analysis of the crude reaction mixture indicated formation of the desired aminal (4*R*,5*R*)‐**6** (Figure 1) along with the over‐reduced primary alcohol product (*R*,*R*)‐**7** (Figure 8), which could correspond to one of the two possible diastereoisomers of the ring‐opened hydroxylated metabolite **M4**. Nonetheless, once more, isolation of either reduction product in pure form was not achieved. Interestingly, both the formation of the primary alcohol (ion at *m/z* 355.0887) as well as the broad peak shape of the extracted ion chromatograph at the *m/z* 353.0730 of (4*R*,5*R*)‐**6** indicated an equilibrium between the desired closed aminal structure of **M3b** (expected as two diastereoisomers) and its open aminoaldehyde form (Figure 8). As additional proof for this equilibration, both the synthetic mixture and a post‐administration sample extract led to the formation and detection of the same hydrazone derivatives upon treatment with Girard's reagent T (data not shown). The above results indicate that, as previously reported [6], (4*R*,5*R*)‐**6** corresponds to a true aminal‐type metabolite of LGD‐4033 (**M3**), coded as **M3b**. However, (4*R*,5*R*)‐**6** differs from the related, previously reported metabolite **M3a** [5, 6] in terms of peak shape, retention time, and product ion mass spectrum (Figure 9). Thus, the latter might correspond to a different hydroxy derivative of LGD‐4033 where the hydroxylation is located at another position of the pyrrolidine ring.

**FIGURE 10:**
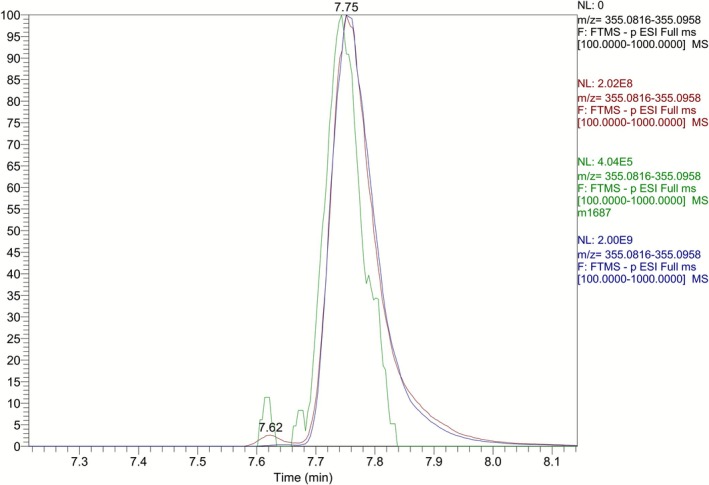
Extracted ion chromatograms at *m/*z 355.0887 [M‐H]^−^ (**M4b**) of a blank sample (in black), a LGD‐4033 post‐administration sample (in red at Day 1; in green at Day 7), and pure synthetic ring‐opened hydroxylated derivative (*R*,*R*)‐**7** (in blue).

**FIGURE 11:**
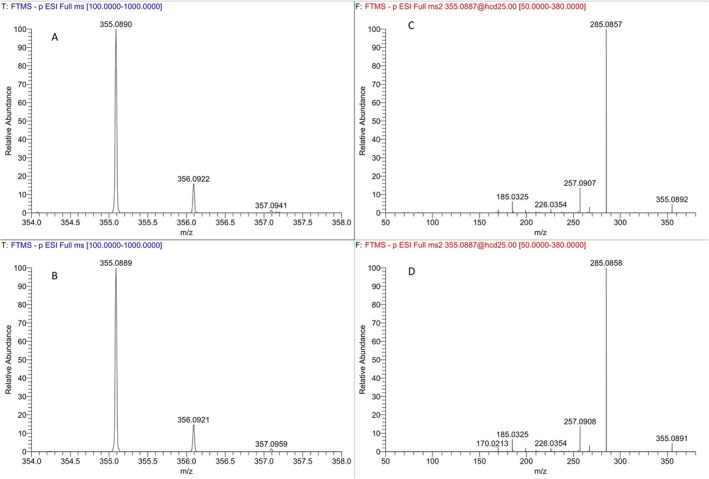
Partial full scan mass spectrum (ESI, negative mode) of (A) **M4b** in a post‐administration urine sample and (B) the synthetic standard (*R*,*R*)‐**7**. Product ion mass spectrum of the deprotonated molecule at *m/*z 355.0887 [M‐H]^−^ (ESI, negative mode) of (C) **M4b** in a post‐administration urine sample and (D) the synthetic standard (*R*,*R*)‐**7**.

Attempts to secure the ring‐opened hydroxylated derivative (*R*,*R*)‐**7** through either further reduction of the above synthetic mixture (NaBH_4_, MeOH) or through direct reduction of carboxylic acid (*R*,*R*)‐**5** (BH_3_·THF, 0°C) were also unsuccessful. Eventually, the (*R*,*R*)‐isomer of the ring‐opened hydroxylated derivative was secured through a two‐step sequence starting from carboxylic acid (*R*,*R*)‐**5** (Figure 7). Thus, treatment of a solution of carboxylic acid (*R*,*R*)‐**5** in a mixture of benzene/MeOH with excess of a 2.0‐M solution of trimethylsilyldiazomethane in diethyl ether, and subsequent reduction of the crude methyl ester thus obtained with LiBH_4_ in THF solution secured the desired primary alcohol derivative (*R*,*R*)‐**7** in 94% yield over two steps. Comparison of the extracted ion chromatograms of the synthetic (*R*,*R*)‐primary alcohol derivative **7** and a post‐administration urine sample (Figure 10) at *m/z* 355 (attributed to **M4**), as well as their partial full scan and product ion spectra (Figure 11) verified that the synthesized alcohol (*R*,*R*)‐**7** corresponds to one of the two possible diastereoisomers of the in vivo observed metabolite **M4**, coded **M4b** and can be used as reference material for this metabolite. Furthermore, this metabolite can be detected in urine samples, up to 9 days post‐administration, employing full scan mode.

### 3.4 Tris‐Hydroxylated Metabolites (**M6**)

In the course of this project, it was proven that hexanoic acids (*R,R*)*‐* **5** and (*R,S*)*‐* **5** correspond to the major (**M5b**) and the minor (**M5a**) metabolites of LGD‐4033, respectively. Consequently, it was reasoned that the diastereoisomers of the related *α*‐hydroxylated hexanoic acids might correspond to some of the three isomeric tris‐hydroxylated metabolites **M6** (Figure 1) at *m/z* 385.0628 that can be observed in post‐administration urine samples [5, 10].

**FIGURE 12:**
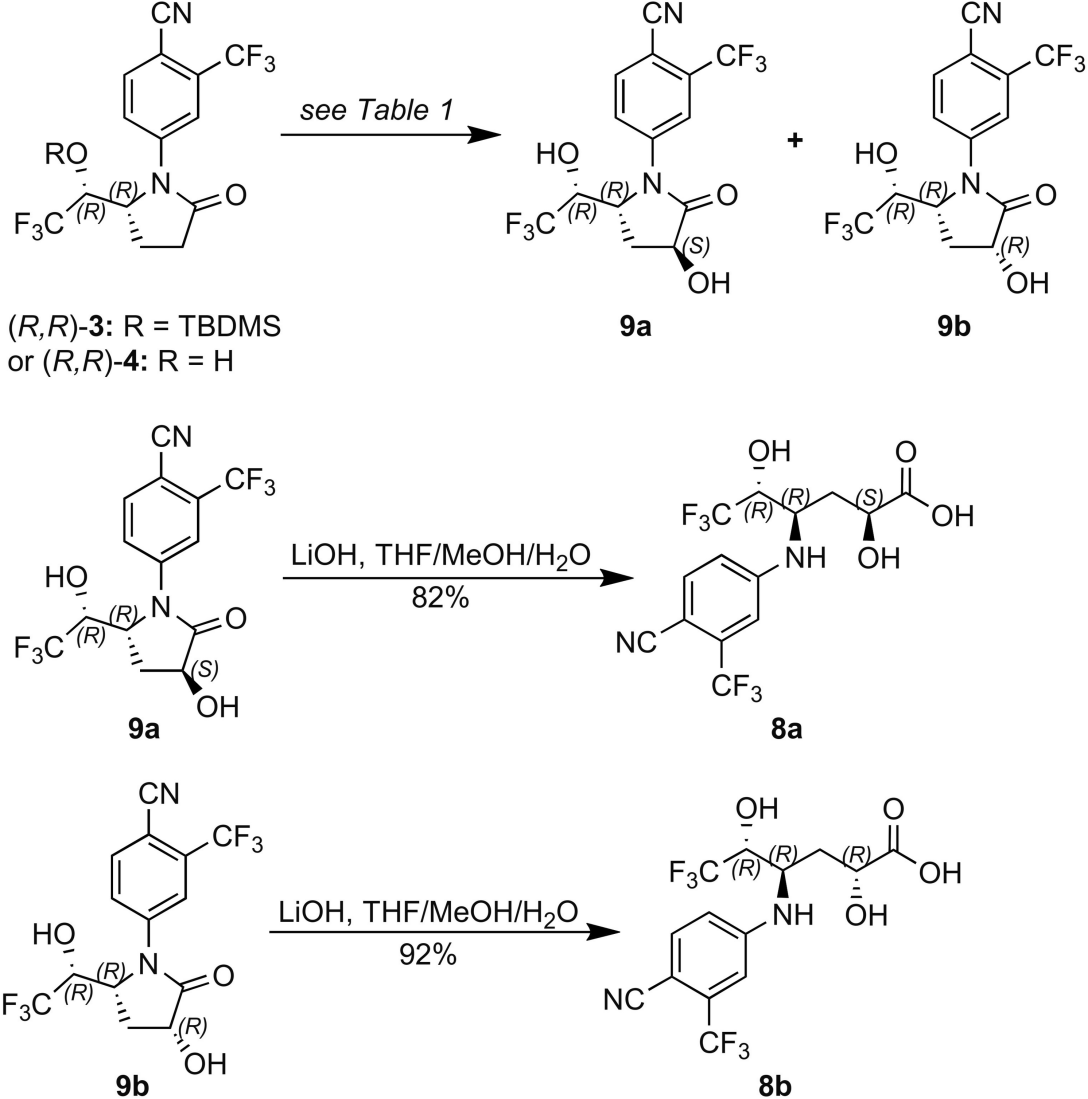
Synthesis of potential **M6**‐related derivatives.

To investigate this possibility, the synthesis and evaluation of the two possible diastereoisomeric *α*‐hydroxylated derivatives of hexanoic acid (*R,R*)*‐* **5** (**8a** and **8b**, Figure 12) were pursued. To this end, (*R*,*R*)‐pyrrolidinones **3** and **4** were exploited as pivotal synthetic intermediates. Treatment of the potassium enolate derived from the silyl ether‐protected lactam (*R*,*R*)‐**3** with Davis' oxaziridine [15] yielded a mixture of diastereoisomeric hydroxylated products (ca. 3:1 ratio), which could be separated upon removal of the silyl ether protective group to provide diols **9a** and **9b** (Figure 12 and Table 1, Entry 1). It should be noted that the stereochemistry of the newly formed stereocenter could not be established unequivocally and the one indicated (i.e., [2*S*] for major product [**9a**] and [2*R*] for the minor one [**9b**]) was tentatively assigned, assuming preferential oxidation of the intermediate enolate from the less hindered *si*‐face. A slightly improved overall yield was achieved by employing oxygen as the oxidant [16] (44%; Table 1, Entry 2 vs. 38%; Table 1, Entry 1). However, the diastereoselectivity of the transformation was diminished (approximately **9a**/**9b** 3:1; Table 1, Entry 1 vs. **9a**/**9b** 1.5:1; Table 1, Entry 1). On the other hand, diol **9b** could be obtained in very good yield and exclusively upon oxygen‐mediated oxidation of the unprotected lactam (*R*,*R*)‐**4** (Figure 12 and Table 1, Entry 3). The exquisite diastereoselectivity of the latter transformation is interesting not just from the purely synthetic point of view but also because it might mimic the in vivo metabolic fate of LGD‐4033. Subsequent alkaline hydrolysis of the lactam ring of **9a** and **9b** secured the corresponding dihydroxyhexanoic acids **8a** and **8b**, respectively (Figure 12).

**TABLE 1:** Attempted oxidations of (*R*,*R*)‐lactams **3** and **4** (Figure 12) and their outcome.

Entry	Lactam	Conditions	Product(s) (% isolated yield)
1	**3**	1. KHMDS (2 eq.), −78°C, THF; PhSO_2_N(O)CPh 2. TBAF, THF	**9a** (29%) + **9b** (9%)
2	**3**	1. LiHMDS (1.5 eq), (MeO)_3_P, O_2_, THF, −78 to −10°C 2. TBAF, THF	**9a** (27%) + **9b** (17%)
3	**4**	1. LiHMDS (1.5 eq), (MeO)_3_P, O_2_, THF, −78 to −10°C	**9b** (87%)

**FIGURE 13:**
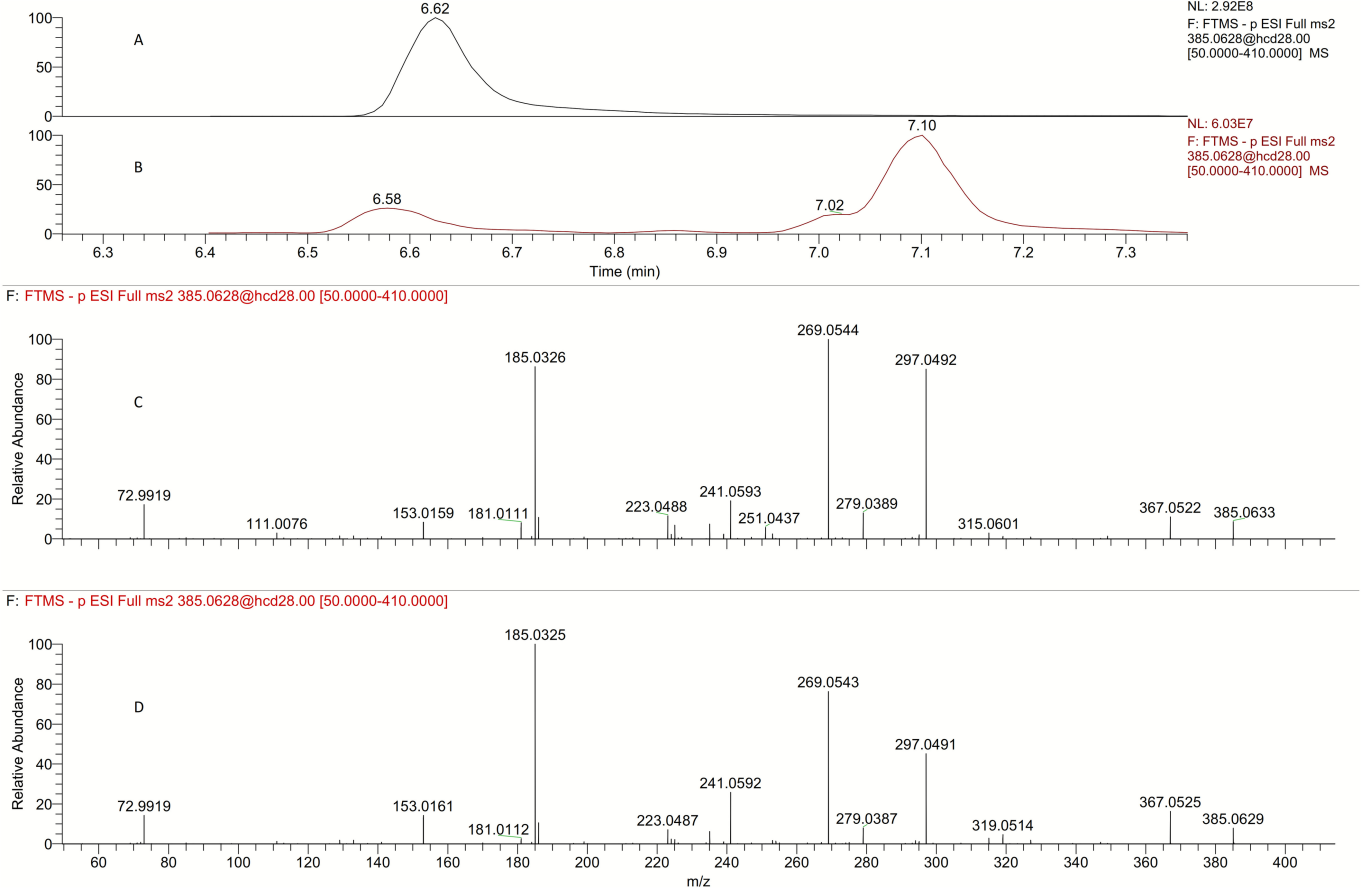
(A) Extracted ion chromatogram of a pure synthetic standard of **8a** at *m/z* 385.0628 [M‐H]^−^. (B) Extracted ion chromatogram of a post‐administration sample at *m/z* 385.0628 [M‐H]^−^. (C) Product ion mass spectrum of pure synthetic standard **8a** at retention time 6.62 min. (D) Product ion mass spectrum of a post‐administration sample at retention time 6.58 min.

**FIGURE 14:**
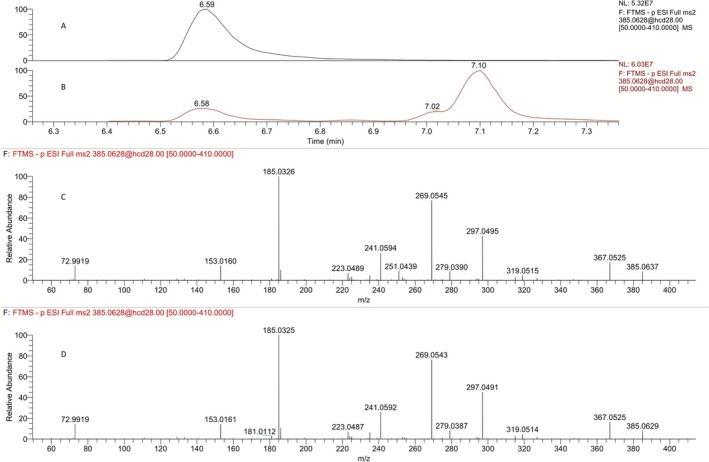
(A) Extracted ion chromatogram of a pure synthetic standard of **8b** at *m/z* 385.0628 [M‐H]^−^. (B) Extracted ion chromatogram of a post‐administration sample at *m/z* 385.0628 [M‐H]^−^. (C) Product ion mass spectrum of pure synthetic standard **8b** at retention time 6.59 min. (D) Product ion mass spectrum of a post‐administration sample at retention time 6.58 min.

Three peaks are observed for the tris‐hydroxylated metabolites (**M6a, M6b**, and **M6c**) of LGD‐4033 in the extracted ion chromatogram of a LGD‐4033 post‐administration urine sample (Figures 13B and 14B). LC–MS analysis of the two synthetic diastereoisomeric products (i.e., **8a** and **8b**) resulted in chromatographic peaks with very similar retention times and very similar mass spectral data. Both **8a** and **8b** (Figures 13A and 14A, respectively) are eluting very close to the first of the related peaks, observed in post‐administration urine samples (Figures 13B and 14B). Hence, based solely on the observed chromatographic retention times the structure of **8a** or **8b** could not be unambiguously assigned to the faster eluting tris‐hydroxylated metabolite of LGD‐4033. To resolve this ambiguity, the relative abundance of the product ions observed in product ion spectra was used as a discrimination tool. Once more, at first glance, very similar spectra were observed for **8a** (Figure 13C), **8b** (Figure 14C), and the faster eluting tris‐hydroxylated metabolite of LGD‐4033 (Figures 13D and 14D). However, a comparison between the relative abundance of the product ions in the spectra of **8a**, **8b**, and the urine‐observed metabolite (Table 2) indicates that the spectrum of **8a** deviates from that of the metabolites while the spectrum of **8b** is in close agreement. Thus, based on the smaller difference between the retention times observed for **8b** versus **8a** with the retention time observed for the faster eluting tris‐hydroxylated metabolite of LGD‐4033 (ΔRt = 0.01 min for **8b** vs. ΔRt = 0.04 min for **8a**) and, mainly, based on the ion relative abundance in their product ion spectra, and in accordance with WADA TD2023IDCR [13], it appears that, overall, compound **8b** (Figure 12) constitutes a better match for the faster eluting tris‐hydroxylated metabolite of LGD‐4033, **M6a**. It is worth noting that the two additional tris‐hydroxylated metabolites of LGD‐4033, eluting after 7.02 and 7.10 min (**M6b** and **M6c**, Figures 13B and 14B), do not correspond to either **8a** or **8b**. These metabolites might correspond to either *α*‐hydroxylated derivative(s) of the epimeric dihydroxylated metabolite [(*R,S*)*‐* **5**] or *β*‐hydroxylated derivative(s) of the main or the minor dihydroxylated metabolite [(*R,R*)*‐* **5** or (*R,*S)*‐* **5**, respectively].

**TABLE 2:** Comparison of the relative intensities of selected ions in the product ion spectra of **8a**, **8b**, and the urine‐observed tris‐hydroxylated metabolite with Rt 6.58 min.

Product ion (*m/z*)	Product ion relative abundance (%)		
	Urine‐observed metabolite (Rt 6.58 min).	**8a** (Rt = 6.62 min)	**8b** (Rt = 6.59 min)
153	13.4	10.2	14.3
185	100	100	100
241	24.6	21.9	26.2
269	77.1	112	75.2
297	46.5	98.5	42.5
367	16.3	12.8	17.8

### 3.5 Evaluation of *α*‐Hydroxy‐Pyrrolidinones **9a** and **9b** as Potential LGD‐4033 Metabolites

**FIGURE 15:**
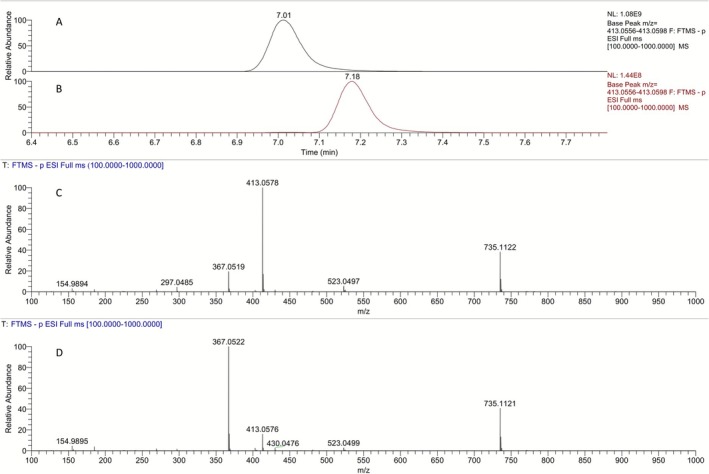
Extracted ion chromatograms in negative ion mode at *m/z* 413.0577 [M + FA‐H]^−^for (A) **9b** and (B) **9a**. Full scan mass spectrum of (C**) 9b** and (D) **9a**.

**FIGURE 16:**
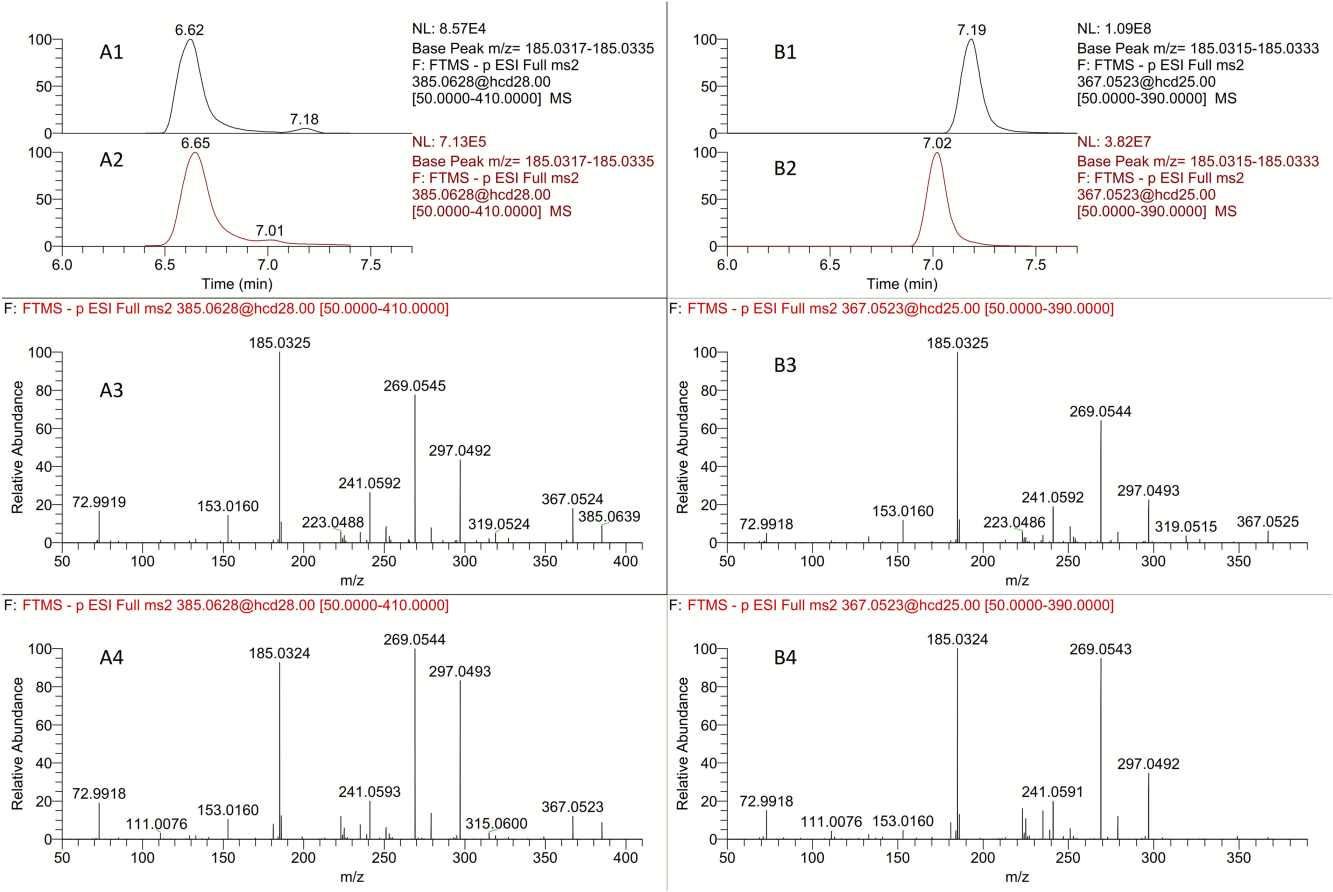
Extracted ion chromatogram of the ion transition of *m/z* 385.0628 [M‐H]^−^ to the product ion at *m/z* 185.0326 for (A1) **8a** and (A2) **8b**. Product ion mass spectra of the precursor ion at *m/z* 385.0628 [M‐H]^−^ for (A3) **8a** and (A4) **8b**. Extracted ion chromatogram of the ion transition of *m/z* 367.0523 [M‐H]^−^ to the product ion at *m/z* 185.0326 for (B1) **9a** and (B2) **9b**. Product ion mass spectra of the precursor ion at *m/z* 367.0523 [M‐H]^−^ for (B3) **9a** and (B4) **9b**.

The availability of the *α*‐hydroxy‐pyrrolidinones **9a** and **9b** (Figure 12) prompted their evaluation under targeted LC‐HRMS analytical methods as potential, previously unreported, metabolic markers of LGD‐4033. To this end, the full scan and PRM mass spectra of **9a** and **9b** were studied and are presented in Figure 15. Two main signals can be observed in their full scan HRMS spectra, with [*M* − H]^−^ at m/z 367.0577 being the predominantly observed ion in the spectrum of **9b**, while the FA at *m/z* 413.0577 is the main ion for **9a**. Interestingly, the PRM of the precursor ion at *m/z* 367.0523 of these synthetic intermediates exhibited high similarity to those of the synthetic standards **8a** and **8b** derived from the precursor ion at *m/z* 385.0628, as shown in Figure 16, suggesting that fragmentation of **8a** and **8b** may proceed through the pyrrolidinone structures **9a** and **9b** produced spontaneously during ESI ionization. The same behavior has been observed for the metabolites **M5a** and **M5b**, which produce the corresponding pyrrolidinones **M2a** and **M2b** during ESI [8, 12].

**FIGURE 17:**
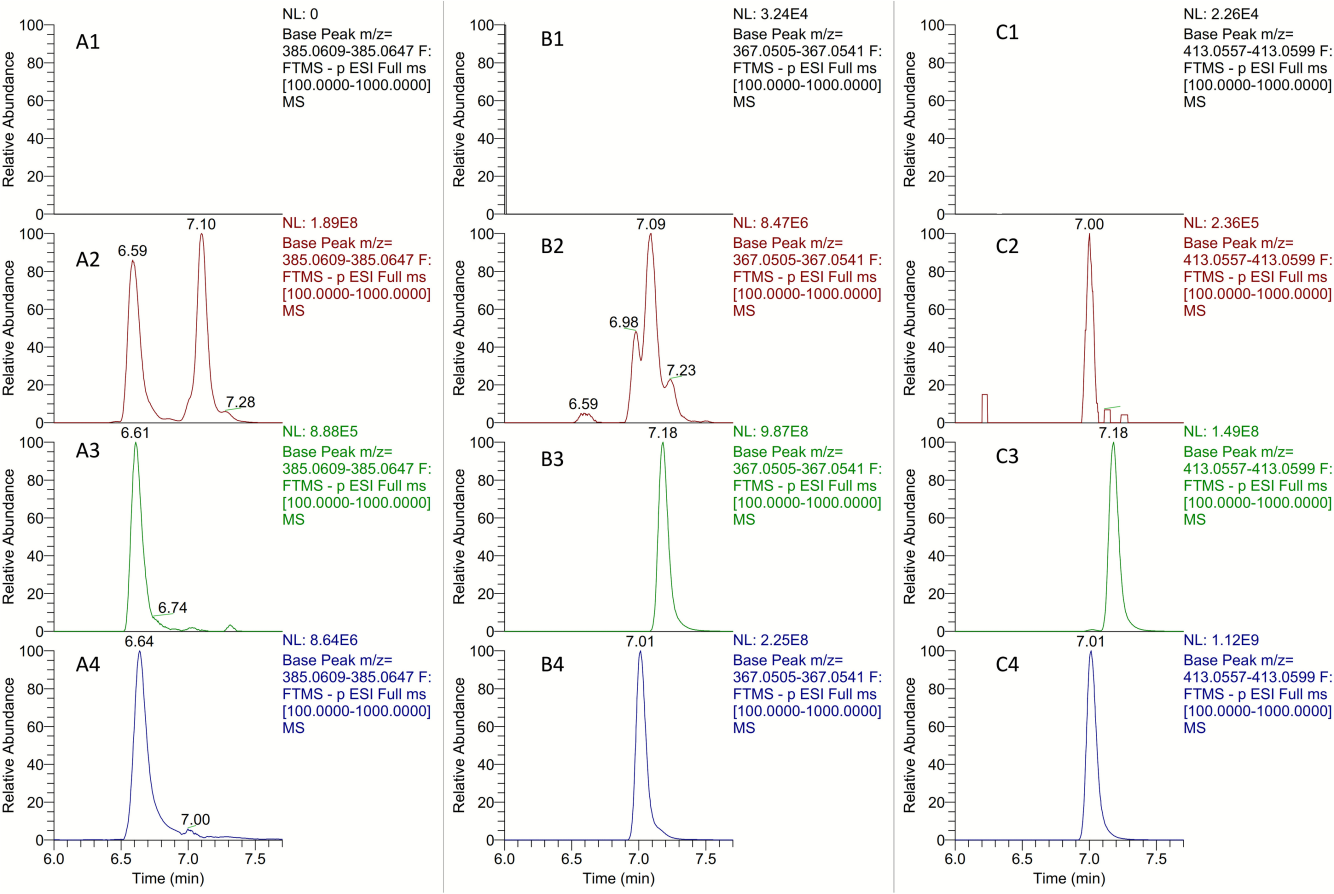
Extracted ion chromatogram at *m/z* 385.0628 [M‐H]^−^ for (A1) blank sample, (A2) a 24 h post‐administration sample, (A3) a synthetic **8b**, and (A4) a synthetic **8a** sample. Extracted ion chromatogram at *m/z* 367.0523 [M‐H]^−^ for (B1) blank sample, (B2) a 24 h post‐administration sample, (B3) a synthetic **9a**, and (B4) a synthetic **9b** sample. Extracted ion chromatogram at *m/z* 413.0577 [M + FA‐H]^−^ for (C1) blank sample, (C2) a 24 h post‐administration sample, (C3) a synthetic **9a**, and (C4) a synthetic **9b** sample.

Nevertheless, post‐administration samples were analyzed against these synthetic standards, and the results are presented in Figure 17. The extraction of the ion at *m/z* 367.0577 resulted in four different peaks in the post‐administration samples, as presented in Figure 17B2. However, the peaks observed at Rt's 6.59‐, 6.98‐, and 7.09‐min coelute with signals observed upon extraction of the ion at *m/z* 385.0628, as shown in Figures 14B and 17A2. Therefore, no conclusive determination can be made as to whether these signals correspond to real LGD‐4033 metabolites or to dehydrated species produced from the ionization of the **M6b** and **M6c** metabolites during ESI. On the other hand, the extraction of the FAs of **9a** and **9b** at *m/z* 413.0577, which are not present in the full scan mass spectra of the up to now synthesized trihydroxylated LGD‐4033 derivatives **8a** and **8b**, resulted in a distinct peak at 7.01 min that corresponds to the structure of **9b**. This signal can be detected in post‐administration samples for 4 days. Hence, structure **9b** can be attributed to a true, previously unreported metabolite of LGD‐4033, **Μ7**. Interestingly, the detection of signals at *m/z* 367.0523 at the same retention time as those obtained at *m/z* 385.0628 suggests that the metabolites **M6b** and **M6c** [5], which are also important for equine and camel doping control [17, 18], are regio‐ or stereo‐isomers of the structures **8a** and **8b**. Further research is required to establish the structures of the remaining two tris‐hydroxylated metabolites. The synthetic sequence developed in the framework of this project could be exploited to this end.

## 4 Conclusion

The structure elucidation of several important LGD‐4033 metabolites was performed and is presented in this study. In more detail, it was shown that (4*R*,5*S*)‐4‐{[4‐cyano‐3‐(trifluoromethyl)phenyl]amino}‐6,6,6‐trifluoro‐5‐hydroxyhexanoic acid ([*R*,*S*]‐**5**, (Figure 2) is, in all respects examined, identical to the minor metabolite of LGD 4033 (**M5a**) and can serve as a reference material for this important metabolic marker. Furthermore, it was concluded that the (4*R*,5*S*)‐isomer of pyrrolidinone **4** (Figure 2) corresponds to the minor pyrrolidinone‐type metabolite of LGD‐4033 (**M2d**). Both metabolites, **M5a** and **M2d**, were detectable up to the last collected sample of the administration study, which was collected 21 days post‐administration, and can thus be considered additional long‐term metabolic markers. Similarly, comparison of synthetic materials with a post‐administration urine sample permitted the complete structural elucidation of the ring‐opened hydroxylated metabolite **M4b** and of one of the urine‐observed tris‐hydroxylated metabolites (**M6a**). Thus, the **M4b** metabolite is the 4‐(((2*R*,3*R*)‐1,1,1‐trifluoro‐2,6‐dihydroxyhexan‐3‐yl)amino)‐2‐(trifluoromethyl)‐benzonitrile) ([*R*,*R*]‐**7**, Figure 7), while the (2*R*,4*R*,5*R*)‐4‐{[4‐cyano‐3‐(trifluoromethyl)phenyl]amino}‐6,6,6‐trifluoro‐2,5‐dihydroxyhexanoic acid) (**8b**, Figure 12) corresponds to the faster eluting **M6a** metabolite. Furthermore, the *α*‐hydroxy‐pyrrolidinone **9b** (Figure 12) was identified as a new short‐term metabolite of LGD‐4033 that was detected in human post‐administration urine, and was coded as **M7**. From the perspective of doping control, the monitoring of **M5a**, as well as both **M2c** and **M2d**, which remain detectable up to the final collected post‐administration sample, is proposed for inclusion in both the initial and the confirmation procedures, complementary to **M5b,** the primary biomarker currently used by the laboratories for the monitoring of LGD‐4033.

## Author Contributions

**Yiannis S. Angelis:** conceptualization, funding, data acquisition, data curation, manuscript writing, manuscript editing. **Panagiotis Sakellariou:** data acquisition, data curation, manuscript writing, manuscript editing. **Mario Thevis:** funding, and manuscript editing. **Andreas Thomas:** data curation, manuscript editing. **Michael Petrou:** funding, manuscript editing. **Emmanuel N. Pitsinos:** funding, conceptualization, synthesis of compounds, data curation, manuscript writing, manuscript editing.

## Funding

The presented work was conducted with the support of the World Anti‐Doping Agency (WADA Research Grant: 21A17EP).

## Conflicts of Interest

The authors declare no conflicts of interest.

## Supporting information


**Data S1:** Supplementary Information.

## Data Availability

The data that support the findings of this study are available from the corresponding author upon reasonable request.
