# Supplementary material for: Further Insights Into the Metabolism of LGD‐4033 in Human Urine. Part 1. Structure Elucidation of Additional Important Metabolites
Source: Drug Test Anal. 2025 Dec 9;18(2):245–59. doi: 10.1002/dta.70009 (PMC12861595; doi:10.1002/dta.70009)
Supplement: Supplementary file 1 — Data S1: Supplementary Information. [file DTA-18-245-s001.docx]

**Supplementary Information**

**Further Insights into the Metabolism of LGD-4033 in Human Urine.
Part 1. Structure Elucidation of Additional Important Metabolites**

Yiannis S. Angelis^[a]^*, Panagiotis Sakellariou^[b]^, Mario Thevis^[b,c]^, Andreas Thomas^[b]^, Michael Petrou^[d]^, Emmanuel N. Pitsinos^[e,f]*^

**1 Materials**

All reagents were purchased at the highest commercial quality and used without further purification, unless otherwise stated. LGD-4033 (CAS Number 1165910-22-4) and *epi*-LGD-4033 (CAS Number 1165910-23-5) were procured from Toronto Research Chemicals.

Stock solutions of the synthesized reference materials were prepared in methanol and stored at −20 °C. Diethyl ether, *n*-pentane and ethyl acetate were of analytical grade and obtained from Labscan (Dublin, Ireland). Dipotassium hydrogen phosphate (K_2_HPO_4_), potassium dihydrogen phosphate (KH_2_PO_4_), disodium carbonate (Na_2_CO_3_), sodium hydrogen carbonate (NaHCO_3_) and sodium sulfate (Na_2_SO_4_) were purchased from Panreac (Barcelona, Spain). Acetonitrile (ACN) for mobile phase preparation was of LC-MS grade and purchased from Riedel-de Haen (Seelze, Germany). Acetic acid, formic acid and Girard reagent T were purchased from Merck (Darmstadt, Germany) and ammonium formate from Acros Organics. HPLC-grade water was obtained by purifying water in a filtration system (Millipore, Billerica, MA, USA). β-glucuronidase from *Escherichia coli* was obtained from Sigma-Aldrich (Steinheim, Germany).

**2 Synthesis of compounds**

**2.1 Synthesis methods**

All reactions were carried out under a dry argon atmosphere with anhydrous solvents (freshly distilled over the appropriate desiccant or dried over 3 Å molecular sieves) under anhydrous conditions, unless otherwise noted. Reactions requiring anhydrous conditions were carried out in oven dried (120 °C, 24 h) or flame dried (vacuum < 0.5 Torr) glassware. All reactions were magnetically stirred with Teflon stir bars, and temperatures were measured externally. All reactions were monitored by Thin Layer Chromatography (TLC) carried out on 0.25 mm E. Merck silica gel plates (60F254). UV light (254 nm) was used for visualization and an acidified ethanolic solution of *p*-anisaldehyde or an acidified aqueous solution of ceric ammonium molybdate and heat were used as developing agents. Compounds were purified on a Biotage^®^ Selekt Enkel Automated flash chromatography unit, equipped with the Spektra software and employing Biotage^®^ Sfär Duo silica columns, or by manual flash column chromatography, employing E. Merck silica gel (60 Å, particle size 0.040–0.063 mm) or Acros Organics silica gel (60 Å, particle size 0.035–0.070 mm). Optical rotations were recorded using a Perkin-Elmer 241 polarimeter at the sodium D line (589 nm) using a 10 cm path-length cell in the solvent and concentration indicated. Infrared (IR) spectra were recorded on a Nicolet 6700 FT-IR spectrometer. Nuclear Magnetic Resonance (NMR) spectra were recorded using a Bruker Avance DRX 500 MHz or a Bruker Avance III 250 MHz instrument and were calibrated using as internal reference the residual nondeuterated solvent for ^1^H-NMR and the deuterated solvent for ^13^C-NMR, respectively (e.g., CDCl_3_: *δ*_H_ = 7.26 ppm, *δ*_C_ = 77.16 ppm; CD_3_OD: *δ*_H_ = 3.31 ppm, *δ*_C_ = 49.00 ppm). Multiplicities are designated as singlet (s), doublet (d), triplet (t), quartet (q), quintet (quint.) or multiplet (m). Broad or obscured peaks are indicated as “br” or “obs”, respectively. To facilitate NMR spectra comparisons, the established LGD-4033 skeleton numbering was used for all compounds when assigning signals. High resolution mass spectra (HRMS) were acquired on a LC qExactive plus HRMS (Thermo Scientific, Bremen, Germany) instrument.

**2.2 Synthetic procedures and characterization data**

**4-[(2*R*)-2-{(1*S*)-1-[(*tert*-butyldimethylsilyl)oxy]-2,2,2-trifluoro­ethyl}­pyrro­lidin-1-yl]-2-(trifluoromethyl)-benzonitrile** [(*R*,*S*)*-***2**]: *epi*-LGD-4033 (58.2 mg, 172 µmol) was placed in a 5 mL pear-shaped flask and imidazole (0.37 g, 5.5 mmol), TBDMSCl (0.41 g, 2.7 mmol), and DMF (0.25 mL) were added sequentially under an argon atmosphere. The mixture was stirred at ambient temperature for 1 week. Volatiles were removed under reduced pressure and the residue was dissolved in dichloromethane (4 mL). Silica gel (1 g) was added, volatiles were removed under reduced pressure, the residue was loaded on top of a chromatography column (silica gel), and eluted with 95:5 → 90:10 *n*-hexane/ethyl acetate to provide TBS-protected *epi*-LGD-4033 (**2**) as amorphous white solid (77.9 mg, 172 µmol; 100% yield).

***R_f_*** *=* 0.14 (silica gel, *n*-hexane/ethyl acetate 95:5); ${\boldsymbol{[}\text{α}\boldsymbol{]}}_{\text{D}}^{\text{25}}$ = +65 (*c* = 0.93, CHCl_3_); **IR** (film) *ν*_max_ = 2957, 2931, 2860, 2222, 1730, 1613, 1556, 1512, 1455, 1381, 1298, 1269, 1175, 1133, 1089, 1072, 1040, 1017, 988, 924, 893, 859, 843, 826, 798, 780, 694, 677 cm^–1^; **^1^H NMR** (500 MHz, CDCl_3_): *δ_H_* = 7.64 (d, *J* = 8.7 Hz, 1 H, H-6), 6.86 (d *J* = 2.5 Hz,, 1 H, H-3), 6.69 (dd, *J* = 8.7, 2.6 Hz, 1 H, H-5), 4.29 (qd, *J* = 7.2, 1.2 Hz,1 H, H-12), 4.17–4.14 (m, 1 H, H-8), 3.53 (ddd, *J* = 9.4, 8.0, 4.2 Hz,1 H, H-11), 3.30 (dt, *J* = 9.4, 7.5 Hz 1 H, H-11′), 2.49 (td, *J* = 8.2, 3.8 Hz, H-9), 2.27–2.21 (m, 1 H, H-10), 2.09–1.95 (m, 2 H, H-9’ & H10’), 0.88 (s, 9 H, (CH_3_)_3_CSi), −0.02 & −0.19 (2 s, 6 H, CH_3_SiCH_3_) ppm; **^13^C NMR** (125 MHz, CDCl_3_): *δ_C_* = 149.0 (s, C-4), 136.4 (s, C-6), 134.6 (q, *J* = 32 Hz, C-2), 124.6 (q, *J* = 285 Hz, C-14), 122.7 (q, *J* = 274 Hz, C-16), 117.0 (s, C-1), 114.1 (s, C-5), 110.1 (s, C-3), 95.7 (s, C-15), 69.4 (q, *J* = 29 Hz, C-12), 58.5 (s, C-8), 49.8 (s, C-11), 25.7 (s, (*C*H_3_)_3_CSi & C-10), 24.5 (s, C-9), 18.1 (s, (CH_3_)_3_*C*Si), −4.6, −5.4 (2 s, *C*H_3_Si*C*H_3_) ppm; **HRMS** (ESI −): *m/z* calculated for C_21_H_27_F_6_N_2_O_3_Si^−^ [*M*+HCOO]^−^: 497.1701, found 497.1689.

**4-[(2*R*)-2-{(1*S*)-1-[(*tert*-butyldimethylsilyl)oxy]-2,2,2-trifluoroethyl}-5-oxo-pyrrolidin-1-yl]-2-(trifluoromethyl)benzonitrile** [(*R*,*S*)*-***3**]: A 25 mL round-bottom flask equipped with an efficient magnetic stirring bar was charged with RuCl_3_ (2.8 mg, 13 µmol) and 10% w/v aqueous NaIO_4_ solution (0.8 mL, 0.4 mmol) was added. To the stirred black solution that ensued was added dropwise a solution of (*R*,*S*)*-***2** (46.7 mg, 103 µmol) in ethyl acetate (2.5 mL), the flask was sealed, and the mixture was vigorously stirred at ambient temperature for 1.5 h. Water (5 mL) and ethyl acetate (10 mL) was added and the organic phase was separated. The aqueous layer was extracted with ethyl acetate (2 x 10 mL). To the combined organic layers was added isopropanol (4 mL), the mixture was stirred for 1 h, and the black precipitate formed was removed by filtration through a short pad of Celite. The filtrate was washed with brine (2 x 15 mL), dried over Na_2_SO_4_, and concentrated. Chromatographic purification (silica gel, *n*-hexane/EtOAc 98:2 to 8:2) gave pyrrolidinone (*R*,*S*)*-***3** as light brown oil (44.7 mg, 95.8 µmol; 93% yield).

***R_f_*** *=* 0.26 (silica gel, *n-*hexane/EtOAc 8:2); ${\boldsymbol{[}\text{α}\boldsymbol{]}}_{\text{D}}^{\text{25}}$ = +16 (*c* = 0.89, CHCl_3_); **IR** (film) *ν*_max_ = 2956, 2932, 2861, 2232, 1715, 1611, 1505, 1473, 1464, 1441, 1383, 1337, 1278, 1175, 1140, 1108, 1071, 1050, 1031, 1004, 970, 914, 879, 831, 782, 671, 604, 553 cm^–1^; **^1^H NMR** (500 MHz, CDCl_3_): *δ_H_* = 8.15 (br s, 1 H, H-3), 7.89–7.85 (m, 2 H, H-5 & H-6), 4.75 (dd, *J* = 9.2, 2.9 Hz, 1 H, H-8), 4.07 (q, *J* = 7.2 Hz, 1 H, H-12), 2.84–2.74 (m, 1 H, H-10), 2.62–2.53 (m, 2 H, H-10′ & H-9), 2.32–2.21 (m, 1 H, H-9′), 0.88 (s, 9 H, (CH_3_)_3_CSi), −0.05 & −0.20 (2 s, 6 H, CH_3_SiCH_3_) ppm; **^13^C NMR** (125 MHz, CDCl_3_): *δ_C_* = 175.3 (s, C-11), 141.3 (s, C-4), 136.0 (s, C-6), 134.3 (q, *J* = 32.8 Hz, C-2), 124.1 (q, *J* = 284.5 Hz, C-14), 123.2 (s, C-5), 122.1 (q, *J* = 272.9 Hz, C-16), 118.9 (q, *J* = 4.9 Hz, C-3), 115.3 (s, C-15), 105.6 (s, C-1), 69.6 (q, *J* = 29.7 Hz, C-12), 58.3 (s, C-8), 31.9 (s, C-10), 25.6 (s, *C*H_3_)_3_CSi), 18.1 (s, (CH_3_)_3_*C*Si), 17.0 (s, C-9), −4.9 & −5.3 (2 s, *C*H_3_Si*C*H_3_) ppm; **HRMS** (ESI +): *m/z* calculated for C_20_H_25_F_6_N_2_O_2_Si^+^ [*M*+H]^+^: 467.1584, found 467.1579.

**4-{(5*R*)-2-oxo-5-[(1*S*)-2,2,2-trifluoro-1-hydroxyethyl]pyrrolidin-1-yl}-2-(trifluoromethyl)benzonitrile** [(*R*,*S*)-**4**]: A solution of TBS-protected pyrrolidinone (*R*,*S*)*-***3** (44.7 mg, 95.8 µmol) in THF (2.0 mL) was treated at ambient temperature and under an atmosphere of argon with 1.0 M solution of TBAF in THF (0.10 mL, 10x10^−5^ mol). After 0.5 h, half saturated aqueous NH_4_Cl solution (5.0 mL) was added and the mixture was extracted with ethyl acetate (3 x 10 mL). The combined organic layers were washed with brine (10 mL), dried over Na_2_SO_4_, and concentrated. Chromatographic purification (silica gel, *n*-hexane/EtOAc 95:5 to 60:40) gave pyrrolidinone (*R*,*S*)-**4** as colorless solid (32.0 mg, 90.8 µmol, 95% yield).

***R_f_*** *=* 0.10 (silica gel, *n*-hexane /EtOAc 8:2); ${\boldsymbol{[}\text{α}\boldsymbol{]}}_{\text{D}}^{\text{25}}$ = +15 (*c* = 1.0, MeOH); **IR** (film) *ν*_max_ = 3407, 2924, 2234, 1691, 1610, 1505, 1442, 1395, 1325, 1277, 1176, 1129, 1051, 1023, 971, 914, 844, 673, 556 cm^–1^; **^1^H NMR** (500 MHz, CD_3_OD): *δ_H_* = 8.32 (d, *J* = 2.2 Hz, 1 H, H-3), 8.05 (d, *J* = 8.5 Hz, 1 H, H-6), 7.89 (dd, *J* = 8.5, 2.3 Hz, 1 H, H-5), 4.92 (ddd, *J* = 8.8, 3.0, 1.4 Hz, 1 H, H-8), 4.07 (q, *J* = 7.5 Hz, 1 H, H-12), 2.81 (ddd, *J* = 17.3, 10.1, 8.7 Hz, 1 H, H-10), 2.54 (ddd, *J* = 17.4, 10.4, 4.0 Hz, 1 H, H-10′), 2.49–2.43 (m, 1 H, H-9), 2.29 (ddt, *J* = 13.2, 10.3, 8.7 Hz, 1 H, H-9′) ppm; **^13^C NMR** (125 MHz, CD_3_OD): *δ_C_* = 177.9 (s, C-11), 143.0 (s, C-4), 137.4 (s, C-6), 134.4 (q, *J* = 32.5 Hz, C-2), 126.5 (s, C-5), 126.1 (q, *J* = 283.1 Hz, C-14), 123.8 (q, *J* = 273.1 Hz, C-16), 122.1 (q, *J* = 5.1 Hz, C-3), 116.3 (s, C-15), 106.6 (br d, *J* = 2.5 Hz, C-1), 69.1 (q, *J* = 29.4 Hz, C-12), 60.1 (s, C-8), 32.9 (s, C-10), 18.7 (s, C-9) ppm; **HRMS** (ESI −): *m/z* calculated for C_15_H_11_F_6_N_2_O_4_^−^ [*M*+HCOO]^−^: 397.0628, found 397.0632.

**(4*R*,5*S*)-4-{[4-cyano-3-(trifluoromethyl)phenyl]amino}-6,6,6-trifluoro-5-hydroxyhexanoic acid** [(*R,S*)*-***5** ]: To a stirred solution of pyrrolidinone (*R*,*S*)-**4** (33.8 mg, 96.0 µmol) in THF/MeOH/H_2_O 6:2:2 (3.0 mL) was added at 0 °C LiOH·H_2_O (15.8 mg, 377 µmol). The mixture was maintained at 0 °C for 30 min and it was then stirred at ambient temperature for 12 h. The reaction was quenched at 0 °C by the dropwise addition of AcOH/H_2_O 1:1 (4 mL). The mixture was diluted with EtOAc (20 mL) and washed with half saturated brine (2 x 10 mL). The aqueous washings were extracted with EtOAc (3 x 5 mL) and the combined organic layers were dried over Na_2_SO_4_, and concentrated under reduced pressure. Benzene (3 x 10 mL) was added to the residue and volatiles were removed under reduced pressure. The light-yellow solid thus obtained was dissolved in acetone (5 mL), silica gel (1.5 g) was added, volatiles were removed under reduced pressure, and the residue was loaded on top of a chromatography column (silica gel, CH_2_Cl_2_/EtOAc 98:2). Elution with 2–40% EtOAc/AcOH (98:2) in CH_2_Cl_2_ provided carboxylic acid (*R,S*)-**5** as colorless glass (33.2 mg, 89.7 µmol, 93% yield).

***R_f_*** *=* 0.21 (silica gel, CH_2_Cl_2_/EtOAc/AcOH 80:20:1); ${\boldsymbol{[}\text{α}\boldsymbol{]}}_{\text{D}}^{\text{25}}$ = +39 (*c* = 1.0, MeOH); **IR** (film) *ν*_max_ = 3351, 2935, 2224, 1710, 1613, 1533, 1449, 1358, 1310, 1272, 1174, 1120, 1100, 1042, 877, 825, 553 cm^–1^; **^1^H NMR** (500 MHz, CD_3_OD): *δ_H_* = 7.60 (d, *J* = 8.7 Hz, 1 H, H-6), 7.03 (s, 1 H, H-3), 6.86 (d, *J* = 8.7 Hz, 1 H, H-5), 4.00–3.91 (m, 2 H, H-8 & H-12), 2.40–2.27 (m, 2 H, H-10 & H-10′), 2.23–2.16 (m, 1 H, H-9), 1.94–1.85 (m, 1 H, H-9′) ppm; **^13^C NMR** (125 MHz, CD_3_OD): *δ_C_* = 176.9 (s, C-11), 153.0 (s, C-4), 137.6 (s, C-6), 135.0 (q, *J* = 31.4 Hz, C-2), 126.6 (q, *J* = 283.3 Hz, C-14), 124.2 (q, *J* = 272.9 Hz, C-16), 118.2 (s, C-15), 114.7 (br s, C-5), 111.6 (br s, C-3), 94.9 (s, C-1), 72.0 (q, *J* = 28.8 Hz, C-12), 52.7 (s, C-8), 31.0 (s, C-10), 26.5 (s, C-9) ppm; **HRMS** (ESI −): *m/z* calculated for C_14_H_11_F_6_N_2_O_3_^−^ [*M*−H]^−^: 369.0679, found 369.0684.

******4-{[(2*R*,3*R*)-1,1,1-trifluoro-2,6-dihydroxyhexan-3-yl]amino}-2-(trifluoro­methyl)benzonitrile** [(*R*,*R*)-**7**]: A stirred solution of hexanoic acid (*R,R*)-**5** (13.1 mg, 35.4 µmol) in MeOH/PhH 1:3 (4.0 mL) was treated at ambient temperature with 2.0 M solution of trimethylsilyldiazomethane in diethyl ether (0.1 mL, 200 µmol). After 15 min, excess reagent was quenched by dropwise addition of a 17% v/v solution of acetic acid in methanol (0.4 mL). CAUTION: gas evolution. The ensuing light-yellow solution was stirred at ambient temperature for 30 min and it was then concentrated under reduced pressure. Benzene (3 x 5 mL) was added to the residue and volatiles were removed under reduced pressure. The methyl ester thus obtained as light-yellow wax was used in the next step without purification.

To an ice cold, stirred solution of the above methyl ester in THF (2.0 mL) was added in one portion LiBH_4_ (11.5 mg, 528 µmol). The mixture was stirred at 0°C for 10 min and then at ambient temperature for 12 h. The reaction was quenched by dropwise addition of 0.1 N aqueous HCl solution (4.0 mL). The mixture was extracted with EtOAc (3 x 5 mL) and the combined organic layers were washed with brine (2 x 5 mL), dried over Na_2_SO_4_, and concentrated under reduced pressure. The residue was purified by chromatography (silica gel, 3% MeOH in CH_2_Cl_2_) to provide diol (*R*,*R*)-**7** as light-yellow oil (11.9 mg, 33.4 µmol, 94% yield).

***R_f_*** *=* 0.08 (silica gel, 3% MeOH in CH_2_Cl_2_); ${\boldsymbol{[}\text{α}\boldsymbol{]}}_{\text{D}}^{\text{25}}$ = +6.5 (*c* = 0.65, MeOH); **IR** (film) *ν*_max_ = 3358, 2943, 2886, 2222, 1614, 1533, 1453, 1360, 1314, 1275, 1175, 1120, 1042, 878, 825 cm^–1^; **^1^H NMR** (500 MHz, CD_3_OD): *δ_H_* = 7.58 (d, *J* = 8.7 Hz, 1 H, H-6), 7.07 (s, 1 H, H-3), 6.88 (d, *J* = 8.7 Hz, 1 H, H-5), 4.06 (q, *J* = 6.8 Hz, 1 H, H-12), 3.97–3.92 (m, 1 H, H-8), 3.57 (t, *J* = 6.4 Hz, 2 H, H-11 & H-11’), 1.83–1.71 (m, 2 H, H-9 & H-9′), 1.68–1.53 (m, 2 H, H-10 & H-10′) ppm; **^13^C NMR** (125 MHz, CD_3_OD): *δ_C_* = 153.4 (s, C-4), 137.5 (s, C-6), 135.0 (q, *J* = 31.5 Hz, C-2), 126.5 (q, *J* = 282.9 Hz, C-14), 124.3 (q, *J* = 272.9 Hz, C-16), 118.3 (s, C-15), 114.6 (br s, C-5), 111.5 (br s, C-3), 94.2 (s, C-1), 71.4 (q, *J* = 29.5 Hz, C-12), 62.6 (s, C-11), 52.8 (s, C-8), 30.0 & 29.9 (2 s, C-10 & C-9) ppm; **HRMS** (ESI −LGM68): *m/z* calculated for C_14_H_13_F_6_N_2_O_2_^−^ [*M*−H]^−^: 355.0887, found 355.0888.

**4-{(5*R*,3*R/S*)-3-hydroxy-2-oxo-5-[(1*R*)-2,2,2-trifluoro-1-hydroxyethyl]­pyrrolidin-1-yl}-2-(trifluoromethyl)benzonitrile** (**9a**/**9b**): To a stirred solution of TBS-protected pyrrolidinone (*R,R*)-**3** (40.1 mg, 86.0 µmol) in THF (2.5 mL) at −78 °C and under an atmosphere of oxygen were added sequentially a 0.42 M solution of (MeO)_3_P in THF (0.20 mL, 84 µmol) and a 1.0 M solution of LiHMDS in THF (0.15 mL, 150 µmol). The mixture was allowed to gradually (over 2 h) warm up to −10 °C. The reaction was quenched by dropwise addition of 0.1 N aqueous HCl solution (2.0 mL) and the mixture was extracted with ethyl acetate (3 x 5 mL). The combined organic layers were washed with brine (10 mL), dried over Na_2_SO_4_, and concentrated. Chromatographic purification (silica gel, 20 to 40% EtOAc in *n*-hexane) gave partially purified hydroxy-pyrrolidinones (32.8 mg), as a mixture of diastereoisomers (ca. 1.5:1, based on ^1^H NMR spectrum). The two diastereoisomers could not be separated by column chromatography. Thus, the mixture was used directly in the next step.

The above mixture was dissolved in THF (2.0 mL) and treated at ambient temperature and under an atmosphere of argon with 1.0 M solution of TBAF in THF (0.10 mL, 10x10^−5^ mol). After 15 min, half saturated aqueous NH_4_Cl solution (5.0 mL) was added and the mixture was extracted with ethyl acetate (3 x 10 mL). The combined organic layers were washed with brine (10 mL), dried over Na_2_SO_4_, and concentrated. Chromatographic purification (silica gel, 30% EtOAc in *n*-hexane to 100% EtOAc) gave 2-hydroxy-pyrrolidinone **9a** as light brown wax (8.6 mg, 23 µmol, 27% yield over two steps) and 2-hydroxy-pyrrolidinone **9b** as light-yellow wax (5.4 mg, 15 µmol, 17% yield over two steps).

2-hydroxy-pyrrolidinone **9a**: ***R_f_*** *=* 0.24 (silica gel, *n*-hexane /EtOAc 4:6); ${\boldsymbol{[}\text{α}\boldsymbol{]}}_{\text{D}}^{\text{25}}$ = +0.4 (*c* = 1.0, MeOH); **IR** (film) *ν*_max_ = 3360, 2923, 2232, 1701, 1611, 1505, 1441, 1396, 1322, 1274, 1176, 1131, 1053, 1000, 964, 897, 843, 678, 562 cm^–1^; **^1^H NMR** (500 MHz, CD_3_OD): *δ_H_* = 8.11 (d, *J* = 2.2 Hz, 1 H, H-3), 7.97 (d, *J* = 8.5 Hz, 1 H, H-6), 7.90 (dd, *J* = 8.5, 2.1 Hz, 1 H, H-5), 4.76 (td, *J* = 7.6, 4.4 Hz, 1 H, H-8), 4.38 (dd, *J* = 7.8, 4.8 Hz, 1 H, H-10), 4.28 (q, *J* = 7.2 Hz, 1 H, H-12), 2.67 (dt, *J* = 14.6, 7.7 Hz, 1 H, H-9), 2.05 (dt, *J* = 14.0, 4.7 Hz, 1 H, H-9′) ppm; **^13^C NMR** (125 MHz, CD_3_OD): *δ_C_* = 176.4 (s, C-11), 145.3 (s, C-4), 136.4 (s, C-6), 133.6 (q, *J* = 32.4 Hz, C-2), 128.9 (s, C-5), 126.1 (q, *J* = 283.1 Hz, C-14), 123.9 (q, *J* = 273.0 Hz, C-16), 123.8 (q, *J* = 5.0 Hz, C-3), 116.4 (s, C-15), 106.8 (br d, *J* = 2.0 Hz, C-1), 74.0 (q, *J* = 29.0 Hz, C-12), 70.7 (s, C-10), 58.2 (s, C-8), 32.1 (s, C-9) ppm; **HRMS** (ESI −): *m/z* calculated for C_14_H_9_F_6_N_2_O_3_^−^ [*M*−H]^−^: 367.0523, found 367.0524.

2-hydroxy-pyrrolidinone **9b**: ***R_f_*** *=* 0.12 (silica gel, *n*-hexane /EtOAc 4:6); **^1^H NMR** (500 MHz, CD_3_OD): *δ_H_* = 8.17 (s, 1 H, H-3), 8.00–7.95 (m, 2 H, H-6 & H-5), 4.94 (dd, *J* = 8.9, 4.1 Hz, 1 H, H-8), 4.76 (dd, *J* = 10.4, 8.1 Hz, 1 H, H-10), 4.57 (br s, 2 H, 2 OH), 4.20 (qd, *J* = 7.7, 3.9 Hz, 1 H, H-12), 2.59 (dd, *J* = 13.0, 8.1 Hz, 1 H, H-9), 2.38–2.31 (m, 1 H, H-9′) ppm; **^13^C NMR** (125 MHz, CD_3_OD): *δ_C_* = 177.3 (s, C-11), 144.8 (s, C-4), 136.7 (s, C-6), 133.9 (q, *J* = 32.6 Hz, C-2), 127.4 (s, C-5), 126.0 (q, *J* = 282.8 Hz, C-14), 123.9 (q, *J* = 273.0 Hz, C-16), 122.5 (q, *J* = 5.0 Hz, C-3), 116.4 (s, C-15), 106.5 (br d, *J* = 2.0 Hz, C-1), 72.3 (q, *J* = 30.1 Hz, C-12), 69.8 (s, C-10), 56.5 (s, C-8), 34.7 (s, C-9) ppm; **HRMS** (ESI −): *m/z* calculated for C_14_H_9_F_6_N_2_O_3_^−^ [*M*−H]^−^: 367.0523, found 367.0525.

**(2*S*,4*R*,5*R*)-4-{[4-cyano-3-(trifluoromethyl)phenyl]amino}-6,6,6-trifluoro-2,5-dihydroxyhexanoic acid** (**8a**): To a stirred solution of hydroxy-pyrrolidinone **9a** (15.9 mg, 43.2 µmol) in THF/MeOH/H_2_O 6:2:2 (2.0 mL) was added at 0 °C LiOH·H_2_O (14.0 mg, 334 µmol). The mixture was maintained at 0 °C for 30 min and it was then stirred at ambient temperature for 12 h. The reaction was quenched at 0 °C by the dropwise addition of AcOH/H_2_O 1:1 (2.0 mL). The mixture was diluted with EtOAc (20 mL) and washed with half saturated brine (2 x 10 mL). The aqueous washings were extracted with EtOAc (3 x 5 mL) and the combined organic layers were dried over Na_2_SO_4_, and concentrated under reduced pressure. Benzene (3 x 5 mL) was added to the residue and volatiles were removed under reduced pressure. To the light-brown oil thus obtained was added methanol (3 x 2 mL) and volatiles were removed under reduced pressure. The residue was washed with dichloromethane (2 x 2 mL) and dried in a vacuum desiccator over P_2_O_5_ to provide dihydroxy-carboxylic acid **8a** as colorless glass (13.7 mg, 35.5 µmol, 82% yield).

***R_f_*** *=* 0.18 (silica gel, CH_2_Cl_2_/EtOAc/AcOH 90:10:1); ${\boldsymbol{[}\text{α}\boldsymbol{]}}_{\text{D}}^{\text{25}}$ = +0.2 (*c* = 0.98, MeOH); **IR** (film) *ν*_max_ = 3352, 2226, 1728, 1615, 1534, 1453, 1357, 1276, 1178, 1137, 1043 cm^–1^; **^1^H NMR** (500 MHz, CD_3_OD): *δ_H_* = 7.58 (d, *J* = 8.7 Hz, 1 H, H-6), 7.14 (d, *J* = 2.4 Hz, 1 H, H-3), 6.96 (dd, *J* = 8.8, 2.5 Hz, 1 H, H-5), 4.26 (dt, *J* = 10.7, 2.9 Hz, 1 H, H-8), 4.10–4.04 (m, 2 H, H-12 & H-10), 2.23 (ddd, *J* = 13.8, 10.8, 2.6 Hz, 1 H, H-9), 1.85 (ddd, *J* = 14.2, 11.1, 3.3 Hz, 1 H, H-9′) ppm; **^13^C NMR** (125 MHz, CD_3_OD): *δ_C_* = 178.0 (s, C-11), 153.7 (s, C-4), 137.3 (s, C-6), 134.8 (q, *J* = 32.2 Hz, C-2), 126.4 (q, *J* = 282.7 Hz, C-14), 124.3 (q, *J* = 272.8 Hz, C-16), 118.3 (s, C-15), 115.2 (br s, C-5), 111.7 (br s, C-3), 94.6 (s, C-1), 71.9 (q, *J* = 29.7 Hz, C-12), 68.3 (s, C10), 49.9 (s, C-8), 38.5 (s, C-9) ppm; **HRMS** (ESI −): *m/z* calculated for C_14_H_11_F_6_N_2_O_4_^−^ [*M*−H]^−^: 385.0628, found 385.0634.

**(2*R*,4*R*,5*R*)-4-{[4-cyano-3-(trifluoromethyl)phenyl]amino}-6,6,6-trifluoro-2,5-dihydroxyhexanoic acid** **(8b)**: To a stirred solution of hydroxy-pyrrolidinone **9b** (12.3 mg, 33.5 µmol) in THF/MeOH/H_2_O 6:2:2 (2.0 mL) was added at 0 °C LiOH·H_2_O (11.2 mg, 267 µmol). The mixture was maintained at 0 °C for 30 min and it was then stirred at ambient temperature for 12 h. The reaction was quenched at 0 °C by the dropwise addition of AcOH/H_2_O 1:1 (2.0 mL). The mixture was diluted with EtOAc (20 mL) and washed with half saturated brine (2 x 10 mL). The aqueous washings were extracted with EtOAc (3 x 5 mL) and the combined organic layers were dried over Na_2_SO_4_, and concentrated under reduced pressure. Benzene (3 x 5 mL) was added to the residue and volatiles were removed under reduced pressure. Chromatographic purification (silica gel, 2% AcOH in acetone/dichloromethane 1:9 to 2% AcOH in acetone/dichloromethane 1:1) of the brown oil thus obtained gave dihydroxy-carboxylic acid **8b** as light brown glass (11.9 mg, 30.8 µmol, 92% yield).

***R_f_*** *=* 0.18 (silica gel, CH_2_Cl_2_/EtOAc/AcOH 90:10:1); ${\boldsymbol{[}\text{α}\boldsymbol{]}}_{\text{D}}^{\text{25}}$ = +0.6 (*c* = 0.55, MeOH); **IR** (film) *ν*_max_ = 3360, 2935, 2224, 1725, 1614, 1533, 1451, 1359, 1275, 1175, 1135, 1043 cm^–1^; **^1^H NMR** (500 MHz, CD_3_OD): *δ_H_* = 7.58 (d, *J* = 8.7 Hz, 1 H, H-6), 7.09 (d, *J* = 2.4 Hz, 1 H, H-3), 6.90 (dd, *J* = 8.7, 2.5 Hz, 1 H, H-5), 4.25–4.14 (m, 3 H, H-8, H-12 & H-10), 2.25–2.17 (m, 1 H, H-9), 2.08–1.99 (m, 1 H, H-9′) ppm; **^13^C NMR** (125 MHz, CD_3_OD): *δ_C_* = 177.2 (s, C-11), 152.8 (s, C-4), 137.3 (s, C-6), 134.8 (q, *J* = 31.6 Hz, C-2), 126.5 (q, *J* = 282.9 Hz, C-14), 124.3 (q, *J* = 272.9 Hz, C-16), 118.3 (s, C-15), 115.1 (br s, C-5), 111.9 (br s, C-3), 94.7 (s, C-1), 70.6 (q, *J* = 29.8 Hz, C-12), 68.4 (s, C10), 49.4 (obs. s, C-8), 37.4 (s, C-9) ppm; **HRMS** (ESI −): *m/z* calculated for C_14_H_11_F_6_N_2_O_4_^−^ [*M*−H]^−^: 385.0628, found 385.0635.
